# A glycoengineered yeast-derived HA1-ferritin nanoparticle vaccine confers protective immunity against H7 avian influenza in chickens

**DOI:** 10.1186/s13567-026-01867-y

**Published:** 2026-10-29

**Authors:** Yutong Liu, Yunlin Shao, Bo Su, Peiqi Liu, Yifeng Huang, Han Zheng, Siqi Xu, Yishan Zhao, Qianyu Li, Ming Liao, Saixiang Feng

**Affiliations:** 1 College of Veterinary Medicine, South China Agricultural University, Guangzhou, 510642 China; 2 State Key Laboratory for Animal Disease Control and Prevention, South China Agricultural University, Guangzhou, 510642 China; 3 Key Laboratory of Zoonoses, Ministry of Agriculture and Rural Affairs, Guangzhou, 510642 China; 4 National and Regional Joint Engineering Laboratory for Medicament of Zoonoses Prevention and Control, Guangzhou, 510642 China; 5 Zhongkai University of Agricultural and Engineering, Guangzhou, 510550 China

**Keywords:** Avian influenza virus, subunit vaccines, *Komagataella phaffii* expression, glycosylation, virus-like particles

## Abstract

H7 subtype avian influenza virus is an important zoonotic pathogen threatening the poultry industry and public health. Recombinant subunit vaccines provide a safer alternative to conventional inactivated vaccines, while yeast expression systems offer advantages of low cost, scalability, and engineering flexibility. However, their application in avian influenza vaccine development remains underexplored. In this study, we use the methylotrophic yeast *Komagataella phaffii* as a low-cost, industrial-scale platform to produce hemagglutinin-1 (HA1) subunits with programmable *N*-/*O*-linked glycosylation. The methanol-utilization-slow (Mut^S^) phenotype strain secreted 1.21 g/L HA1, and compared with the standard methanol-utilization-plus (Mut^+^) phenotype strain, the *N*-linked and *O*-linked glycans were significantly reduced [liquid chromatography–mass spectrometry (LC–MS)/MS glycopeptide profile]. Despite hypoglycosylation, the antigen retained native conformation, binding H7-specific mAb Q74 with a *K*_*D*_ of 1.34 × 10^–7^ M. SpyTag/SpyCatcher-directed conjugation to ferritin drove quantitative self-assembly into 30 nm virus-like nanoparticles. Formulated with CpG-ODN 1826, the HA1-Mut^S^-ferritin candidate elicited Hemagglutination inhibition (HI) titers of 6.7 log_2_ in specific pathogen-free (SPF) leghorns at 28 days post-immunization (dpi), significantly enhancing HA1-specific IgY responses (*p* < 0.0001) while sustaining high-level circulating CD4^+^ T cells. At 28 dpi, chickens were challenged with 10^6^ embryo infectious dose (EID)_50_ of H7N9 avian influenza virus; the formulation conferred 100% clinical protection and accelerated viral clearance, matching the performance of conventional inactivated vaccines. Yeast-based processes significantly reduce downstream purification costs and eliminate reliance on chicken embryos, providing a scalable and biosafe platform for rapid avian influenza control.

**Supplementary information:**

The online version contains supplementary material available at https://doi.org/10.1186/s13567-026-01867-y.

## Introduction

Avian influenza virus (AIV) is an important zoonotic pathogen, with highly pathogenic (HPAIV) strains such as H5 and H7 subtypes causing devastating economic losses to the poultry industry and posing a persistent pandemic threat to human health [1, 2]. These viruses frequently breach species barriers, necessitating the development of robust and scalable vaccine platforms to ensure global biosecurity. Among the viral components, the hemagglutinin (HA) surface glycoprotein—specifically the HA1 subunit—is the primary target for vaccine design due to its pivotal role in receptor binding [3]. Hemagglutinin is one of the most important surface glycoproteins of the influenza virus, responsible for binding to host cell receptors. The HA consists of two subunits: HA1 and HA2 [3, 4]. However, the immunogenicity and functional integrity of HA1 are profoundly influenced by its glycosylation patterns, which modulate viral immune evasion and host infection [5]. While recombinant subunit vaccines offer a safer alternative to conventional inactivated vaccines, their widespread adoption is often hindered by production costs and molecular heterogeneity [6]. The methylotrophic yeast *K. phaffii* has emerged as a robust platform for recombinant protein production and a promising expression system for vaccine manufacturing owing to its cost-effectiveness and scalability; yet, the impact of yeast-specific glycosylation on AIV antigen properties remains underexplored.

The biosafety risks inherent in producing traditional inactivated vaccines are driving a paradigm shift toward safer subunit vaccines, facilitated by advances in recombinant protein expression. This transition is exemplified by recent successes in vaccines for Rotavirus, severe acute respiratory syndrome coronavirus 2 (SARS-CoV-2), and human papillomavirus [7–10]. However, applying these subunit vaccines to enveloped viruses remains challenging, primarily because their surface antigens rely on complex glycosylation modifications [11]. Correct glycosylation is critical for antigens to adopt their native conformation and maintain effective immunogenicity [12]. While mammalian systems represent the industry standard due to their capacity for authentic post-translational modifications (PTMs) and correct glycosylation—which ensure native-like protein structures—their broad application is constrained by high production costs [13, 14]. Conversely, baculovirus–insect cell systems enable high-level expression of eukaryotic proteins with partial PTMs, offering operational simplicity and high productivity [15]. Although widely used for structural biology and virus-like particle (VLP) vaccines, the discrepancies in glycosylation patterns compared with mammalian cells may compromise immunogenicity and stability. Amid these options, *K. phaffii* has emerged as a versatile eukaryotic host that offers the dual advantages of high-yield expression typical of microbial expression systems and the capacity for post-translational modification. Furthermore, its lack of endotoxin production offers a significant biosafety advantage over *E. coli*. Collectively, the system’s scalability and cost-effectiveness make it an ideal platform for the mass production of vaccine antigens [16, 17].

Glycoengineering strategies such as the deletion of endogenous genes encoding glycosyltransferases (e.g., *OCH1*) and introduction of heterologous enzymes may be employed for tailoring *K. phaffii* to produce humanized glycoproteins [18]. Notably, *OCH1*-knockout GlycoSwitch^®^ strains of *K. phaffii* such as SuperMan5, SuperMan5 His^−^, SuperMan5 pep4^−^, SuperMan5 (aox1^−^, Mut^S^), and SuperMan5 (pep4, prb1^−^/sub2^−^) have successfully yielded high-quality recombinant human ceruloplasmin, IFN-α2b, lactoferrin, and rotavirus capsid proteins [10, 19–21]. The use of SuperMan5 is particularly beneficial for expressing glycoproteins with specific glycosylation requirements, as it helps control the glycosylation pattern and prevents excessive glycosylation, which is crucial for maintaining the structural integrity and improving the immunogenicity of glycoproteins. Despite these advancements, there are very few reports on the use of yeast expression systems, especially *K. phaffii*, in the development of vaccines for glycoproteins such as those in influenza. Addressing this gap is of both scientific and practical importance.

Ferritin has emerged as a highly attractive scaffold for vaccine design owing to its unique cage-like nanostructure and intrinsic biological functions [22]. Each ferritin nanoparticle is composed of 24 identical or homologous subunits and safely stores iron, exhibits intracellular bioavailability, and mitigates oxidative stress, thereby protecting cells from damage [23]. Its stable nanoscale architecture has been harnessed for enhancing the antigen presentation and immunogenicity of nanoparticle-based vaccines [24–26]. Complementing carriers, the SpyTag–SpyCatcher system offers a robust strategy for antigen conjugation. Originating from the bacteriophage AP205, SpyTag and SpyCatcher spontaneously form irreversible isopeptide bonds, enabling the assembly of highly stable and immunogenic nanoparticles [27]. This system has been shown to substantially improve antigen-specific immune responses and has been employed in multiple studies for generating safe and potent nanoparticle vaccines [27–29]. This technology has been widely applied in influenza vaccine development, particularly in baculovirus-based expression systems; however, the employment of the SpyTag/SpyCatcher system in yeast expression systems has been sparsely studied and warrants further exploration.

The *K. phaffii* SuperMan5-10 (Δ*OCH1*) strain was used to produce the HA1 subunit of H7 avian influenza virus (AIV), which was then assembled with ferritin into VLPs via the SpyTag/SpyCatcher system. The particle size, glycosylation profile, and antibody binding affinity of the resulting VLPs were thoroughly characterized. Formulation with the adjuvant CpG generated a subunit vaccine that elicited robust humoral and cellular immune responses under in vivo conditions, with ferritin and CpG enhancing antigen-specific immunity and antibody titers, respectively. These findings validate the modular nanoparticle based on ferritin and SpyTag/SpyCatcher technologies for influenza vaccine design. The combination of structural fidelity achieved in *K. phaffii* and the robust protective efficacy observed in vivo establishes this formulation as a promising vaccine candidate for the prevention and control of AIV outbreaks.

## Materials and methods

### Strains, virus, and SPF chickens

Strains of *K. phaffii* (SuperMan5; BioGrammatics, USA), X-33 (ThermoFisher Scientific, USA), and *E. coli* (New England Biolabs, USA) were cultured in BMGY and BMMY medium (Sangon Biotech, China) and Luria–Bertani broth (ThermoFisher Scientific, USA), respectively. The H7 subtype AIV (H7–E229 strain), which belongs to the Yangtze River Delta lineage A [30], was utilized in this study. The corresponding HI test antigen was sourced from our laboratory stocks. Fourteen-day-old specific pathogen-free (SPF) chickens were obtained from Xinxing Dahua Poultry Co., Ltd. (China). All experiments involving infectious viruses were performed in a biosafety level 3 laboratory or animal biosafety level 3 facilities in accordance with protocols approved by the South China Agricultural University (CNAS BL0011). All animal experiments performed herein adhered to the guidelines specified by the Animal Care and Use Committee of South China Agricultural University (2017A002) and were subjected to institutional review and approval (approval no. 2022F166).

### Construction of recombinant plasmids and generation of expression systems

The amino acid sequences of HA1 from influenza A virus A/goose/Guangdong/Q38/2017 (H7N9; GenBank accession no. AUD54811.1) and ferritin (GenBank accession no. WP_000917208.1) were retrieved from the National Center for Biotechnology Information database (NCBI). To prevent unintended proteolytic cleavage during expression, the C-terminal sequence of HA1 was modified by removing two cysteine residues. Codon optimization was carried out to enhance expression from human-preferred codons (Additional file 16). The 5′ and 3′ termini of the gene encoding HA1 were modified to include recognition sites for *Eco*RI and *Xba*I, respectively, while recognition sites for *Eco*RI and *Bam*HI were introduced into the gene encoding ferritin. Synthesized fragments (Sangon Biotech, China) were cloned into the vector pUC19. HA1 and pPICZαA were subjected to double digestion with the restriction enzymes *Eco*RI and *Xba*I, while ferritin and pET28 were subjected to digestion with *Eco*RI and *Bam*HI. Following digestion, the purified fragments were ligated at 37 °C for 1 h and used for transformation of the *E. coli* strain DH5α. The plasmids so obtained were verified by sequencing. All primers used herein are listed in Additional file 17. The recombinant plasmid pET28–ferritin–SpyCatcher with the correct insert sequence was subsequently employed for the transformation of *E. coli* BL21 (DE3), a prokaryotic expression strain. The transformed cells were plated on LB agar containing kanamycin and incubated for 18–24 h at 37 °C. Selected single colonies were verified via polymerase chain reaction (PCR) analysis. Similarly, the verified pPICZαA–H7HA1–SpyTag plasmid was subjected to digestion with *Sac*I and *Bgl*II at 37 °C for 1 h, and the linearized fragment was recovered using a gel extraction kit. The fragment was then used for transformation of the eukaryotic expression strain SuperMan5 using a *K. phaffii* chemical transformation kit and plated on yeast extract-peptone-dextrose (YPD) agar containing zeocin. The plates were incubated at 30 °C for 48–72 h, after which individual colonies were selected and verified via PCR analysis. The constructed strains are listed in Additional file 18.

For analysis of the methanol utilization phenotype, the constructed yeast strains were diluted with 50 µL sterile phosphate-buffered saline (PBS), and 10-µL aliquots were spotted onto MD and MM (with yeast nitrogen base, glucose, and biotin as main components for MD, and yeast nitrogen base, methanol, and biotin for MM) agar plates. The plates were air-dried for 10 min in a biosafety cabinet before incubation at 30 °C for 48 h. Colony morphology and growth were observed; the methanol-sensitive strains exhibited normal growth on methanol-containing media, whereas methanol-insensitive strains exhibited weak or no growth. The methanol-sensitive (Mut^+^) and methanol-insensitive (Mut^S^) strains were subsequently transferred to YPD agar for further culturing.

### Protein expression and purification in *E. coli* and *K. phaffii*

Single colonies of PCR-verified *E. coli* clones were inoculated into LB broth containing kanamycin and cultured at 37 °C under rotary conditions (200 rpm) until an absorbance at 600 nm (OD_600_) value of approximately 0.2 was attained. The recombinant and control cultures were induced with isopropyl β-d-1-thiogalactopyranoside (1.0 mM) and incubated at 16 °C under rotary conditions (200 rpm) for 16 h for low-temperature expression. Following induction, the cells were harvested and suspended in lysis buffer (250 mM Tris, pH 8.5, 300 mM sodium chloride [NaCl], and 20 mM imidazole), followed by disruption at a 4 °C using an ultrasonic homogenizer. The lysates were centrifuged at 4 °C for the removal of cell debris. The supernatants were subsequently loaded onto a Ni-Sepharose Excel column connected to an ÄKTA protein purification system (Cytiva, Sweden). The column was equilibrated with lysis buffer prior to sample loading, and nonspecifically bound proteins were removed with lysis buffer. The bound His-tagged proteins were eluted stepwise or with a linear gradient of imidazole. Protein elution was monitored by measuring absorbance at 280 nm, and the fractions containing the target protein were collected and concentrated by ultrafiltration. Buffer exchange or desalting was performed using a desalting column connected to the ÄKTA system, and the protein so obtained was stored in the desired storage buffer (PBS, pH 7.4 and 10% glycerol).

For the yeast expression system, single colonies of white color and smooth appearance were picked up from YPD agar plates and used for the inoculation of BMGY liquid medium. The culture was incubated overnight until an OD_600_ of 1.5 was attained. The culture was then diluted with BMMY medium such that an initial OD_600_ of 0.6 was attained, followed by incubation at 30 °C under rotary conditions (250 rpm). Methanol (0.5% v/v) was added every 24 h as a carbon source over a 72-h induction period. After induction, the culture was centrifuged, and the supernatant was collected. Secreted proteins were purified using a Ni-affinity column on the ÄKTA system following the same protocol as above for the steps of binding, washing, elution, and buffer exchange. Protein expression and identity were verified using sodium dodecyl sulfate–polyacrylamide gel electrophoresis (SDS-PAGE) and western blot analyses. Protein concentration was determined using the Bradford method, with bovine serum albumin (BSA) serving as the standard for generating standard curves alongside the dialyzed samples. The absorbance was recorded at 595 nm, and protein concentrations were determined using the corresponding standard curve.

### Analysis of the glycosylation profile of HA1-Mut^S^ and HA1-Mut^+^ via liquid chromatography–mass spectrometry (LC–MS)/MS analysis

The purity and molecular weights of the recombinant proteins were first analyzed via SDS-PAGE. Samples were mixed with 5 × loading buffer, denatured in a boiling water bath for 5 min, and loaded onto a 12% resolving gel for SDS-PAGE analysis. Electrophoresis was initially conducted at 80 V for 15 min and then at 120 V for approximately 60 min. After electrophoresis, the gels were stained with Coomassie Brilliant Blue for 30 min and then destained until a clear background was obtained. Target protein bands were excised from the gel and cut into pieces of ~1-mm^3^ volume for subsequent peptide preparation. The gel pieces were destained with 50% acetonitrile/50 mmol L^−1^ Tris–HCl (pH 8.0) and dehydrated with 100% acetonitrile. Disulfide bonds were reduced with 10 mmol L^−1^ dithiothreitol at 56 °C for 1 h and alkylated with 55 mmol L^−1^ iodoacetamide under dark conditions for 1 h. After drying, the gel pieces were digested with chymotrypsin, trypsin combined with Asp-N, or Glu-C as per the experimental design. The enzymes were diluted with Tris–HCl or ammonium bicarbonate buffer, preincubated at 4 °C for 40 min, and used for digestion at 37 °C for 16 h. Peptides were extracted twice with 5% trifluoroacetic acid/50% acetonitrile, pooled, and subjected to vacuum drying. The resulting peptides were further purified using self-packed C18 desalting columns and dried for subsequent LC–MS/MS analysis. The peptide samples were separated on a C18 nano-LC column using 0.1% formic acid in water (A) and 0.1% formic acid in 80% acetonitrile (B) as the mobile phases applied at a flow rate of 300 nL min^−1^ and a 120-min gradient. MS analysis was performed on an Orbitrap platform, with MS1 resolution set to 60 000 (scan range 350–1500 *m*/*z*) and MS2 resolution set to 15000. Collision energy was set at 30 eV. The data were queried against the target protein database using Byonic software, with precursor mass tolerance of 20 ppm and fragment mass tolerance of 0.02 Da, allowing up to three missed cleavage sites. Fixed modifications included carbamidomethylation of cysteine, while variable modifications included methionine oxidation, *N*-terminal acetylation, and *N*-linked and *O*-linked glycosylation. The structural prediction of HA1 glycosylation sites was performed using the Alphafold 3 version 3.0.1 (based on Google ColabFold). Data visualization was performed using PyMOL software.

### Indirect analysis of the binding of HA1-Mut^S^ and HA1-Mut^+^ to H7 monoclonal antibody using ELISA

High-binding 96-well microplates were coated with enzyme-linked immunosorbent assay (ELISA) coating buffer. The test proteins were added to the first row and subjected to twofold serial dilutions with PBS across the plate, leaving the last row as a blank control. The plates were incubated overnight at 4 °C. After the coating step, the wells were blocked with 3% BSA at 26 °C for 1 h. The V5-tagged H7 avian influenza nanobody Q74 (unpublished) was used as the primary antibody. The plates were then incubated at 26 °C for 2 h. The culture plate was then washed five times with washing buffer, for 3 min each time. Mouse anti-V5 tag monoclonal antibody (ab27671; Abcam, UK) served as the secondary antibody, and the plates were incubated for 3 h at 26 °C following its addition. Horseradish peroxidase (HRP)-conjugated goat anti-mouse IgG (100 µL per well; ab6789; Abcam, UK) was used as the tertiary antibody, and the plates were incubated for 1 h. After the washing steps, 100 µL of the substrate 3,3′,5,5′-Tetramethylbenzidine (TMB; ab171523; Abcam, UK) was dispensed into each well, followed by incubation at 37 °C for 30 min under dark conditions. The reaction was terminated by adding an equal volume of TMB stop solution. The absorbance at 450 nm was recorded using Varioskan LUX multimode plate reader (ThermoFisher Scientific, USA).

### SPR characterization of the interaction of HA1-Mut^S^ with H7 monoclonal antibody

Protein–protein binding kinetics were measured using a Biacore 8K surface plasmon resonance (SPR) system (GE Healthcare, Sweden). Prior to the experiment, HA1-Mut^S^ was diluted to 0.5 mg/mL with PBS, and the H7-specific monoclonal nanobody Q74 was diluted to 1 mg/mL. The running buffer was prepared from a 10 × stock solution of HBS-EP (0.1 M HEPES, pH 7.4, 1.5 M NaCl, 30 mM ethylenediaminetetraacetic acid [EDTA], and 0.5% v/v surfactant P20), which was diluted to 1 × concentration with deionized water. The buffer was degassed by sonication prior to use. The buffer and waste bottles were placed according to instrument requirements, and the system was primed for baseline stabilization. A Series S NTA sensor chip was preactivated with nickel ions (Ni^2+^) prior to sample injection. His-tagged HA1-Mut^S^ was immobilized on the chip surface via His–Ni^2+^ coordination at levels of approximately 800–1000 RU to ensure an adequate signal-to-noise ratio for subsequent kinetics measurements. The chip was then washed with running buffer to eliminate nonspecific adsorption. The antibody Q74 was subjected to serial dilutions of 1:400, 1:800, 1:1600, and 1:3200 in running buffer. The samples corresponding to the various concentrations were sequentially injected into the flow cell using multicycle kinetics mode. The association and dissociation phases were monitored for 120 and 300 s, respectively, at a flow rate of 30 µL min^−1^. Following each measurement cycle, the sensor chip was regenerated with 250 mM EDTA to eliminate any remaining ligands and analytes. Sensorgrams were analyzed using Biacore 8K Evaluation Software version 3.2. The data were fitted to a 1:1 Langmuir adsorption model to determine the association rate constant (k_a_), dissociation rate constant (k_d_), and *K*_*D*_.

### Assembly and purification of HA1-Mut^S^-ferritin fusion nanoparticles

To assemble the HA1-Mut^S^-ferritin fusion nanoparticles, an assembly buffer (20 mM Tris–HCl, pH 8.0, and 50 mM NaCl) was prepared and filtered using a 0.22-µm membrane to remove particulates. Purified ferritin-SpyCatcher was then mixed with ELISA-verified HA1-Mut^S^-SpyTag at molar ratios of 1:1, 1:2, and 1:3 for ascertaining the conditions associated with optimal conjugation efficiency. The mixture was adjusted to a final volume of 1 mL with deionized water and incubated overnight at 4 °C to promote covalent SpyTag/SpyCatcher isopeptide bond formation, resulting in the self-assembly of the fusion nanoparticle HA1-Mut^S^-ferritin. Following incubation, the reaction products were analyzed by SDS-PAGE to assess conjugation efficiency and confirm complete assembly. To further purify the assembled nanoparticles and remove unbound monomeric components, the samples were purified using size-exclusion chromatography (SEC). The eluate containing target proteins was concentrated to a volume of 500 µL using a centrifugal filter device (50 kDa molecular weight cutoff; UFC805096; EMD Millipore) and loaded onto a Superose 6 Increase 10/300 GL column (29091596; Cytiva, Sweden) equilibrated with 20 mM Tris–HCl (pH 7.4) and 150 mM NaCl. Fractions containing the target proteins were analyzed by SDS-PAGE, and the molecular weight of the assembled particles was estimated via comparison with protein molecular weight standards.

### Determination of particle sizes of the VLPs via zeta potential measurement

To analyze the surface charge characteristics of HA1-Mut^S^, ferritin, and HA1-Mut^S^-ferritin nanoparticles, the samples were dialyzed against PBS and adjusted to concentrations of approximately 1 mg/mL. To minimize interference due to ionic strength, the samples were diluted with low-ionic-strength PBS (10 mM, pH 7.4) or deionized water prior to measurement and centrifuged to remove any small aggregates or air bubbles. The treated samples were loaded onto disposable electrophoretic cells, ensuring air bubbles were not trapped. Zeta potential measurements were performed at 25 °C using ZetaView (Particle Metrix, Germany). Particle electrophoretic mobility under an applied electric field was monitored via phase analysis light scattering and converted to Zeta potential values (mV). Each sample was measured independently at least three times, and mean values ± standard deviation (SD) were reported to assess the stability and dispersibility of the protein nanoparticles based on their surface charges.

### Determination of particle sizes of the VLPs via DLS analysis

To evaluate the hydrodynamic properties and dispersion status of the protein particles, samples of the proteins HA1-Mut^S^, ferritin, and HA1-Mut^S^-ferritin were dialyzed extensively against PBS and adjusted to a uniform concentration of 1 mg/mL. Prior to measurement, the samples were centrifuged to remove potential aggregates and allowed to equilibrate to eliminate air bubbles. Each protein sample was taken in a clean quartz cuvette, ensuring uniform distribution within the measurement path. Dynamic light scattering (DLS) measurements were conducted on a Zetasizer Pro instrument (Malvern Panalytical, UK) at a constant temperature, and fluctuations in scattered light intensity caused by Brownian motion of the particles were monitored. The autocorrelation data were processed using the instrument software, and the intensity, *Z*-average hydrodynamic sizes, and polydispersity index were derived via cumulant analysis.

### Morphological analysis of VLPs via TEM

To examine the morphological features of the protein particles, samples of purified HA1-Mut^S^, ferritin, and HA1-Mut^S^-ferritin were analyzed via transmission electron microscopy (TEM; Hitachi HT7800/HT7700, Japan). Each sample was mixed with 2% potassium dichromate (negative stain) at a volume ratio of 1:1. Approximately 10 µL was applied onto 200-mesh carbon-coated copper grids (ZXBR, China) and allowed to adsorb for 1–2 min. Any excess liquid was removed, and additional negative stain was applied to enhance contrast. To preserve protein structure, the samples were prefixed with 0.1% glutaraldehyde for 10 min and rinsed with deionized water prior to staining. The samples were subsequently dehydrated using a graded ethanol series (30%~100%) and embedded in epoxy resin, which was cured at 60 °C. Ultrathin sections of ~70–90 nm were cut using an ultramicrotome and mounted on copper grids. The sections were stained with 2% phosphotungstic acid (pH 7.0) and allowed to air-dry before observation. TEM imaging was performed to capture the structural morphology of the protein particles.

### Vaccine preparation and animal immunization

The proteins HA1-Mut^S^, ferritin, and HA1-Mut^+^ were successfully expressed and purified as described above, followed by the conjugation of HA1-Mut^S^ with ferritin. CpG adjuvant (Class B CpG ODNs) was purchased from InvivoGen (tlrl-2007-1, USA). For the inactivated virus control, allantoic fluid containing H7 AIV strain E229 was treated with 0.1% formaldehyde at 37 °C for 48 h, followed by dialysis to remove residual formaldehyde. Complete inactivation was confirmed via three successive passages in 10-day-old embryonated SPF eggs and the lack of any associated detectable hemagglutination activity. The inactivated virus antigen was stored at −80 °C until further use. Concentrations of the HA1-Mut^S^, HA1-Mut^+^, and ferritin samples were adjusted to 0.5 mg/mL. The following immunization doses were used for the various groups: each chicken of (1) the HA1-Mut^+^ and HA1-Mut^S^ groups received 30 µg of protein, and (2) the HA1-Mut^S^-ferritin and HA1-Mut^S^-ferritin-CpG groups received a total of 60 µg of protein, with those of the HA1-Mut^S^-ferritin-CpG group receiving an additional 500 µg of CpG adjuvant. Antigen solutions for all the vaccine (HA1-Mut^+^, HA1-Mut^S^, HA1-Mut^S^-ferritin, and HA1-Mut^S^-ferritin-CpG), inactivated H7–E229 virus, and PBS control groups were emulsified with SEPPIC MONTANIDETM ISA 71VG oil adjuvant (SEPPIC, France) at a 1:1 (v/v) ratio to prepare water-in-oil emulsified vaccines.

A total of 50 SPF white leghorn chickens (female:male ratio of 1:1) were randomly assigned to six groups (control, H7–E229, HA1-Mut^+^, HA1-Mut^S^, HA1-Mut^S^-ferritin, and HA1-Mut^S^-ferritin-CpG), with ten birds per group. Immunizations were carried out via multiple subcutaneous administrations in the neck region. The control and H7–E229 groups received 0.12 mL of emulsified PBS and 0.2 mL of inactivated H7–E229 virus vaccine, respectively, while the HA1-Mut^+^ and HA1-Mut^S^ groups each received 0.12 mL of the corresponding vaccine per bird. The HA1-Mut^S^-ferritin and HA1-Mut^S^-ferritin-CpG groups each received 0.24 mL of the corresponding vaccine. Beginning a week post-immunization, blood samples were collected every 7 days, and serum was separated for antibody analysis until antibody titers declined. At 28 days post-immunization (dpi), chickens were intranasally challenged with 1 × 10^6^ EID_50_ (in 200 μL) of HPAIV H7–E229 and monitored for clinical signs and survival over the following 14 days. To assess viral shedding, oropharyngeal and cloacal swabs were collected on days 3, 5, 7, 9, and 11 post-challenge (dpc), and the virus was reisolated in SPF chicken embryos. Additionally, to evaluate the vaccine’s protective effect against viral-induced lung injury, lung tissues were harvested from three chickens per group on 3 dpc. The samples were fixed in 4% histocellular fixative (Servicebio, China) and embedded in paraffin. Lung sections (5-μm thick) were stained with hematoxylin and eosin (H&E), and histological alterations were examined using a double-blind approach, with representative images taken via optical microscopy.

### Analysis of HA1-specific antibodies using ELISA

HA1-Mut^S^ was dialyzed against PBS and coated onto 96-well ELISA plates at a concentration of 1 µg/well, followed by overnight incubation at 4 °C. After removing the coating solution, the wells were blocked with 300 µL of 3% BSA at 37 °C for 1 h to prevent nonspecific binding. Following blocking, 40 µL of PBS was added to each well, followed by the addition of 10 µL of the serum samples. The plates were incubated at 37 °C for 2 h. After washing the plates three times with PBST (PBS containing Tween‑20), 100 µL of HRP-labeled goat anti-chicken IgG (1:5000; ab97135; Abcam, UK) was added to each well, followed by incubation at 37 °C for 45 min. Following another three washes, 100 µL of the substrate TMB was added and allowed to react for 30 min under dark conditions at 37 °C. The reaction was halted by the addition of 100 µL of ELISA stop solution (C1058; Solarbio, China), and absorbance was measured at 450 nm. The same principle was applied for antibody isotyping; different antigens were coated onto the wells of the microplate, followed by the sequential addition of serum samples and HRP-labeled secondary antibodies or antigen to allow the formation of the antigen–antibody–enzyme complexes. After thorough washing, TMB substrate was added, and absorbance was measured at 450 nm. The concentrations of the antibodies IgY and IgM were calculated based on standard curves using commercial ELISA kits (IgY and IgM ELISA kits, ab157693 and ab157692, respectively; Abcam, UK).

### HI assay

A 96-well V-bottomed plate was used for the HI assay. Columns 1–11 were filled with 25 µL PBS, while column 12 was filled with 50 µL PBS. Chicken serum samples (25 µL) were added to column 1, followed by thorough mixing and twofold serial dilutions across columns 2–10. After mixing, 25 µL of the mixture in column 10 was discarded. Columns 11 and 12 served as controls without serum. Next, 25 µL of inactivated H7 subtype AIV H7–E229 antigen (4 hemagglutination units) was added to the wells in columns 1–11 and incubated at 26 °C for at least 30 min; antigen was not added to column 12. Subsequently, 25 µL of 1% red blood cells was gently added to each well (columns 1–12), mixed carefully, and incubated at 26 °C for 40 min. Hemagglutination patterns were then observed to determine the HI titers.

### Flow cytometry analysis of T cells

Using sterile 0.55-µm needles, peripheral blood was collected from the wing vein of chickens into vacuum tubes containing heparin. Blood was mixed with sterile PBS at an appropriate ratio, and peripheral blood mononuclear cells (PBMCs) were isolated via density gradient centrifugation. The isolated PBMCs were counted and adjusted to a concentration of 1 × 10^5^ cells/mL. Aliquots (100 µL) of the PBMC suspension were placed in 1.5-mL tubes and incubated with appropriately diluted fluorophore-conjugated antibodies (Mouse Anti-Chicken CD3-APC, Mouse Anti-Chicken CD4-FITC, and Mouse Anti-Chicken CD4-PE; SouthernBiotech, USA). All procedures were performed on ice under dark conditions to minimize fluorescence quenching. Following a 20-min incubation on ice, the cells were pelleted by centrifugation at 350 × *g* for 5 min, and the supernatant was removed. The cells were washed twice with PBS containing 2% fetal bovine serum (FBS) to remove unbound antibodies and finally resuspended in 200 µL PBS containing 2% FBS for immediate analysis on CytoFLEX flow cytometer (Beckman Coulter, USA). Data were analyzed with FlowJo version 10.6.2 (FlowJo LLC, USA). The lymphocytes were identified by their forward-scatter (FSC) and side-scatter (SSC) signatures, and a gate was applied to exclude debris and dead cells. Within the CD3^+^ T cell population, CD4^+^ and CD8^+^ T cell subsets were further gated to determine their relative proportions, enabling the evaluation of immune status and T cell distribution.

### Detection of cytokines in PBMC cultures using ELISA

Following their isolation as described above, the PBMCs were suspended in RPMI 1640 medium (Gibco, USA) supplemented with 2% FBS at a concentration of 1 × 10^5^ cells per mL. Two milliliters of cell suspension were added to each well of a six-well plate to ensure uniform distribution. The HA1-Mut^S^ antigen was added to each well at a final concentration of 3 µg/mL, while control wells either did not receive any antigen or received an equal volume of PBS. The plates were incubated at 37 °C in an atmosphere containing 5% carbon dioxide for 24 h to allow antigen-specific cellular responses. Cytokine concentrations were then measured using commercial ELISA kits according to the manufacturers’ protocols: IFN-γ (ab273261; Abcam, UK), IFN-α (ab273260; Abcam, UK), IL-4 (ECH0044; FineTest, China), and TNF-α (ECH6394; FineTest, China). Cytokine concentrations in each sample were quantified based on standard curves.

### Statistical analysis

All experiments were independently conducted a minimum of three times (*n*  ≥  3), and the results have been presented as mean ± SD. Data processing and statistical analyses were performed using GraphPad Prism 9.3 software (GraphPad Software, USA). Differences between two groups were assessed using a two-tailed unpaired Student’s *t*-test. Statistical significance was denoted as follows: ^*^p < 0.05, ^**^p < 0.01, and ^***^p < 0.001.

## Results

### Purification of influenza hemagglutinin HA1 subunit and ferritin

**Figure 1:**
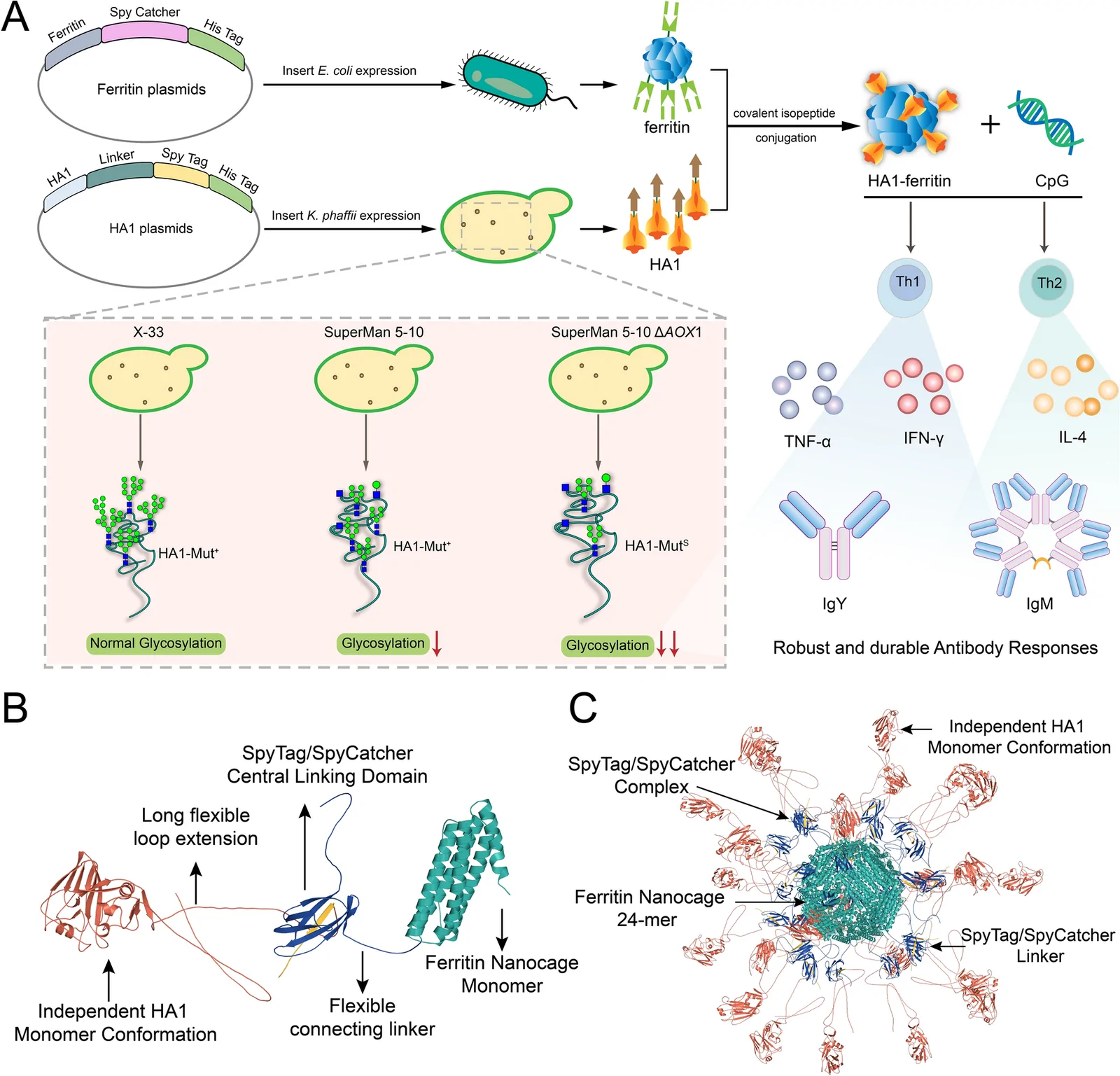
**Schematic of HA1–ferritin nanovaccine construction and the elicited immune responses.** **A** SpyCatcher-tagged ferritin is expressed in *E. coli*, while SpyTag-fused influenza HA1 antigen is produced in *K. phaffii*. HA1 is then directionally conjugated to ferritin nanoparticles via covalent isopeptide bonding mediated by the SpyTag/SpyCatcher system to form HA1–ferritin; coadministration with the CpG adjuvant promotes Th1/Th2-type cytokine responses (TNF-α, IFN-γ, IL-4) and durable antibody responses (IgY, IgM). The lower panel compares the effects of different *K. phaffii* strains (X-33, SuperMan 5–10, and SuperMan 5–10 Δ*AOX1*) on HA1 glycosylation levels. **B** Structural modeling of a single recombinant HA1-ferritin protomer. The HA1 antigen monomer (red) is conjugated to a ferritin subunit (green) via the SpyTag/SpyCatcher system. **C** Simulated 3D conformation of the fully assembled 24-mer HA1-ferritin nanoparticle. The model illustrates the high-density multivalent display of independent HA1 monomers arrayed on the surface of the ferritin nanocage core.

**Figure 2:**
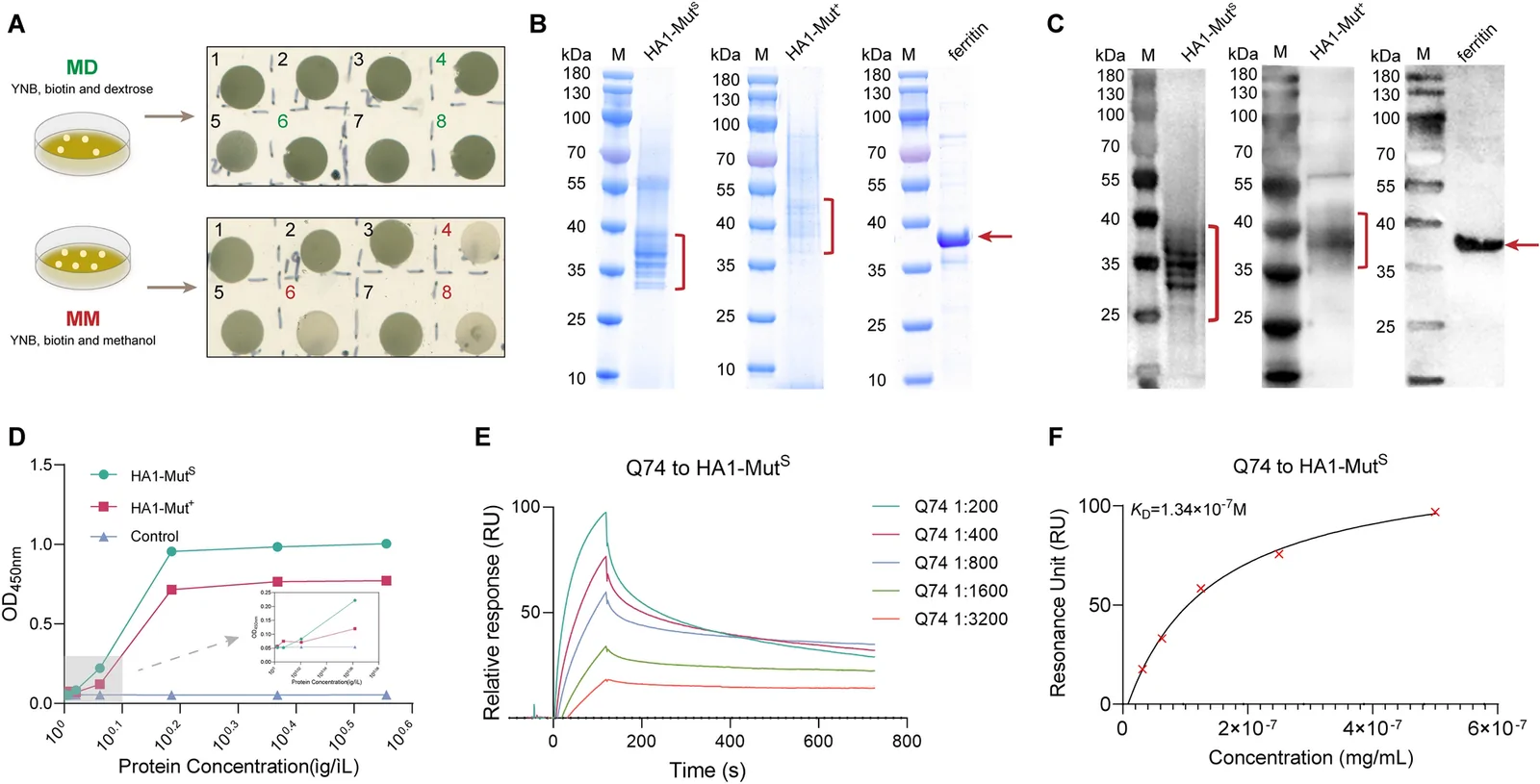
**Expression of HA1 and ferritin proteins and affinity identification of HA1.** **A** Screening of *K. phaffii* Mut^S^ and Mut^+^ strains using MM and MD plates, with strains 4, 6, and 8 representing Mut^S^, and the remaining strains as Mut^+^. **B** SDS-PAGE analysis for preliminary identification of HA1-Mut^S^ (30–40 kDa), HA1-Mut^+^ (35–45 kDa), and ferritin (38 kDa) protein expression. **C** Western blot using His antibody to confirm the expression of HA1-Mut^S^, HA1-Mut^+^, and ferritin proteins. **D** ELISA affinity analysis of HA1-Mut^S^, HA1-Mut^+^, and H7-specific monoclonal antibody Q74, showing stronger antibody recognition for HA1-Mut^S^. **E**, **F** Surface plasmon resonance measurement of binding kinetics for HA1-Mut^S^, HA1-Mut^+^, and H7-specific monoclonal antibody Q74. The equilibrium dissociation constant (*K*_D_) for HA1-Mut^S^ binding to antibody Q74 was 1.34 × 10^−7^ M.

Previous studies have demonstrated that immunization with influenza HA1 antigen can induce robust and specific antibody responses in mice [31]. Here, the HA1 antigen was produced in the glycoengineered *K. phaffii* SuperMan5-10 (Δ*OCH1*) strain and subsequently conjugated to ferritin nanoparticles via the SpyTag/SpyCatcher system to generate an H7 subtype–targeting vaccine (Figure 1A). To visualize this design, structural models of the independent HA1 monomer and the fully assembled 24-mer nanoparticle are presented in Figure 1B and C, respectively. HA1 with low levels of mannose glycosylation was obtained upon expression in the yeast *K. phaffii*. Methanol utilization-deficient (Mut^S^) strains of the yeast were obtained by screening colonies on MD and MM plates containing glucose and methanol as the sole carbon source, respectively. Yeast colonies 4, 6, and 8, harboring the HA1 expression vector, grew slowly and appeared translucent on MM plates, indicating insensitivity to the methanol-induced glycosylation pathway, while the remaining colonies exhibited the ability to utilize methanol (Mut^+^; Figure 2A). The expression of HA1 in the Mut^S^ and Mut^+^ strains was analyzed via SDS-PAGE and western blot. The purified protein HA1-Mut^S^ displayed clear and uniform multiple bands distributed in 30–40 kDa, consistent with its theoretical molecular weight (Figure 2B, C). By contrast, the protein HA1-Mut^+^ exhibited diffuse staining without any distinct band, suggestive of higher levels of glycosylation. SDS-PAGE revealed uniformly diffuse bands in the HA-X33 between 70 and 100 kDa, indicating a higher degree of HA1 glycosylation expressed by the *K. phaffii* strain X33, a widely used host for the high-level expression of recombinant proteins (Additional file 1). The protein ferritin was detected at approximately 38 kDa, which is consistent with the expected molecular weight, thereby confirming successful expression. The concentrations of purified proteins were determined to be 1.21, 0.96, and 1.45 g/L for purified HA1-Mut^S^, HA1-Mut^+^, and ferritin, respectively, using Bradford assay. These results indicate that by screening *K. phaffii* strains insensitive to methanol-induced glycosylation pathways, HA1-Mut^S^ with uniform structure and low glycosylation levels can be efficiently produced. Additionally, the constructed strains efficiently expressed ferritin and may thus serve as a reliable resource for the subsequent assembly of HA1-Mut^S^-ferritin nanoparticle vaccines.

The binding capacities of HA1-Mut^S^ and HA1-Mut^+^ to HA1-specific antibody was preliminarily evaluated via ELISA using the conformational epitope-specific antibody Q74 as the primary antibody. The results demonstrated that an increase in antigen concentration led to a concomitant and gradual increase in the values of absorbance at 450 nm (OD_450_) for both HA1-Mut^S^ and HA1-Mut^+^; however, the magnitude of increase was significantly higher with HA1-Mut^S^ than with HA1-Mut^+^ (Figure 2D). The *K. phaffii*-harboring empty vector did not exhibit any statistically significant change in OD_450_, confirming the reliability of the assay. These results indicate that both HA1-Mut^S^ and HA1-Mut^+^ specifically interact with the conformational epitope-specific antibody Q74, with HA1-Mut^S^ exhibiting stronger antibody recognition. To quantitatively estimate binding affinity, SPR analysis was performed. The antibody Q74 bound to the immobilized target protein on the sensor chip, generating a concentration-dependent response signal measured in resonance units (RU; Figure 2E). A decrease in the concentration of antibody Q74 led to a corresponding decline in RU values, which is consistent with concentration-dependent binding behavior. Using a steady-state fitting model, the equilibrium dissociation constant (*K*_*D*_) for the binding of HA1-Mut^S^ with antibody Q74 was determined to be 1.34 × 10^−7^ M (Figure 2F). These findings confirm that HA1-Mut^S^ binds the cognate antibody with high specificity and affinity, with binding strength increasing concomitantly with rising antibody concentration. Collectively, these results demonstrate that HA1-Mut^S^ and HA1-Mut^+^ are both effective antigens, with HA1-Mut^S^ displaying superior antibody binding affinity, providing a reliable basis for subsequent immunological evaluations.

### Analysis of glycosylation status of recombinant HA1-Mut^S^and HA1-Mut^+^ using mass spectrometry (MS) analysis

**Figure 3:**
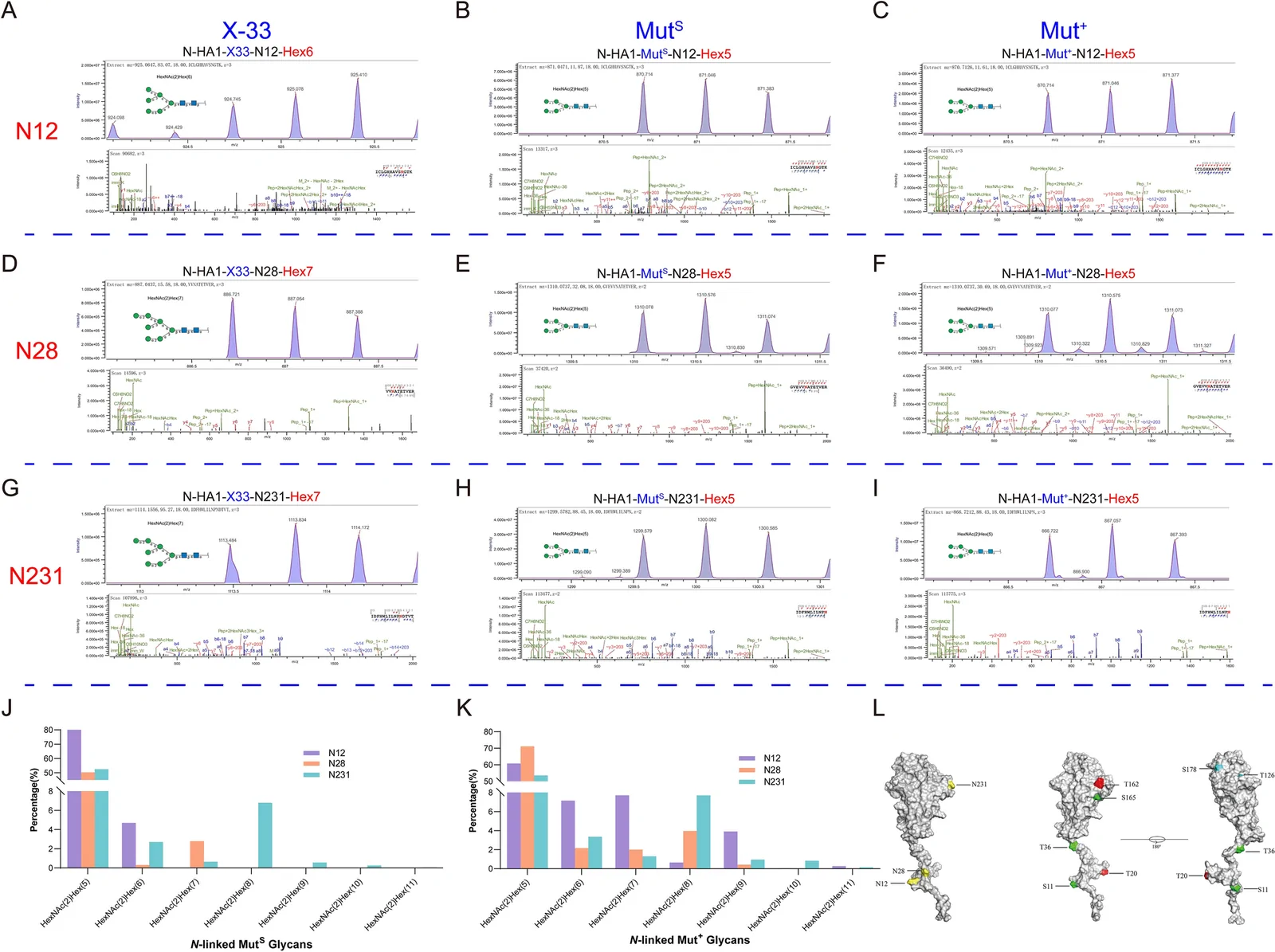
***N*-linked glycosylation analysis and structural modeling of HA1-Mut**^**S**^ **and HA1-Mut**^**+**^. **A**–**C** Mass spectrometric analysis of the *N*-linked glycan population at N12 in HA1-X33, HA1-Mut^S^, and HA1-Mut^+^. **D**–**F** *N*-linked glycosylation mass spectrometric spectra at N28 for the three differentially glycoengineered HA1 proteins. **G**–**I** Glycan profiles at N231, completing the site-specific mapping of the three canonical *N*-linked glycosylation sequons. In HA1-X33, the predominant glycoforms are Hex_6_ at N12 and Hex_7_ at both N28 and N231, whereas HA1-Mut^S^ and HA1-Mut^+^ uniformly present Hex_5_ as the dominant oligosaccharide at all three positions. **J**, **K** Distribution and proportion of major glycan species at the *N*-linked glycosylation sites N12, N28, and N231 in HA1-Mut^S^, HA1-Mut^+^, and HA1-X33 samples. HA1-Mut^+^ primarily contains high-mannose type glycans (HexNAc_2_Hex_7–9_) at these sites, while HA1-Mut^S^ predominantly shows low-mannose type simple glycans (HexNAc_2_Hex_5_). **L** Structural model of HA1 predicted by AlphaFold 3.0. The model shows the location of the glycosylation sites (N12, N28, N231) and their associated glycan modifications (depicted in different colors). This model illustrates the spatial arrangement of the glycosylation sites and their relation to the overall HA1 protein structure.

Comprehensive glycosylation profiling of the proteins HA1-Mut^+^, HA1-Mut^S^, and HA1-X33 was carried out via liquid chromatography–tandem MS (LC–MS/MS). A total of 864 glycans and 3 *N*-linked as well as 37 *O*-linked sites of glycosylation were identified in the case of HA1-Mut^+^ (Additional file 2). By contrast, only 468 glycans were identified in the case of HA1-Mut^S^, corresponding to 3 and 28 sites of*N*-linked and *O*-linked glycosylation, respectively (Additional file 4). Owing to the limited detection capacity for peptides with molecular weights exceeding 5 kDa, the highly glycosylated region of HA1-X33 could not be precisely quantified. A comparative analysis of the dominant glycoforms showed that both HA1-Mut^+^ and HA1-Mut^S^ shared three conserved sites for *N*-linked glycosylation (N12, N28, and N231), which were primarily modified with high-mannose type of glycans (Additional file 2). The *N*-glycans have been categorized as high-mannose, hybrid, or complex types based on the number of mannose residues. High-mannose type of glycans were the predominant type, accounting for 80.48, 79.78, and 67.84% of the modifications at N12, N28, and N231, respectively, in HA1-Mut^+^, and 90.47, 53.39, and 63.51%, respectively, in HA1-Mut^S^. Notably, the proportion of high-mannose glycans at N12 increased by 9.99% in HA1-Mut^S^ compared with that in HA1-Mut^+^ but decreased by 26.39% and 4.33% at N28 and N231, respectively. Both the proteins were expressed under identical conditions; these differences are therefore unlikely to result from variability in proteolysis or expression. Further analysis revealed distinct differences in glycan composition between the two mutants. HA1-Mut^S^ exhibited a comparatively narrow glycan spectrum than HA1-Mut^+^, with HexNAc_2_Hex_5_ and HexNAc_2_Hex_6_ identified at N12, and HexNAc_2_Hex_5_, HexNAc_2_Hex_6_, and HexNAc_2_Hex_7_ detected at N28. By contrast, HA1-Mut^+^ displayed a broader diversity of *N*-glycans ranging from HexNAc_2_Hex_5_ to HexNAc_2_Hex_11_ (Additional file 2). At the *N*-linked glycosylation site N12, HA1-X33 (Figure 3A), HA1-Mut^S^ (Figure 3B), and HA1-Mut^+^ (Figure 3C) exhibit Hex_6_, Hex_5_, and Hex_5_ glycoforms, respectively. At N28 and N231, HA1-X33 shows Hex_7_ (Figure 3D, G), while HA1-Mut^S^ (Figure 3E, H) and HA1-Mut^+^ (Figure 3F, I) predominantly display Hex_5_. These findings indicate that HA1 expressed in X33 cells is hyper-mannosylated with extended glycans (Hex_12_–Hex_16_), which is similar to typical feature of insect cell expression systems. Conversely, the HA1 expressed in the SuperMan5 strain (Mut^S^ and Mut^+^) of *K. phaffii* exhibits a truncated glycosylation profile, in which all three sequons terminate at the Man_5_ core (HexNAc_2_Hex_5_). This truncation is attributed to the loss of endogenous mannosidase I (MAN1) activity, which prevents mannose chain elongation. Therefore, the simplified glycan structure reduces steric hindrance to the HA1 epitope of avian influenza virus, which may enhance immune recognition of the antigen while reducing serum clearance and minimizing the risk of hypersensitivity reactions. An analysis of glycan composition further revealed that HA1-Mut^S^ predominantly contained simple low-mannose glycans (HexNAc_2_Hex_5_; Figure 3J), whereas HA1-Mut^+^ contained high-mannose type glycans (HexNAc_2_Hex_7–9_), which is typical of yeast-derived glycoproteins (Figure 3K). To further understand how these glycan modifications are accommodated in the protein structure, the spatial configuration of glycosylation sites on HA1 was subsequently investigated. To further visualize the *N*-linked and *O*-linked glycosylation sites, the three-dimensional structures of HA1-Mut^+^ and HA1-Mut^S^ were predicted using AlphaFold 3.0, which allowed the mapping of these glycosylation sites onto the protein surface (Figure 3L). The structural models so obtained revealed three conserved sites for *N*-linked glycosylation (N12, N28, and N231) in the exposed loop regions of the molecule. These residues are readily accessible to solvents and are likely to serve as major sites for *N*-linked glycosylation, contributing to proper protein folding and structural integrity. In addition, several putative sites for *O*-linked glycosylation—S11, T20, T36, T126, T162, S165, and S178—were identified. However, their surface distribution was relatively sparse compared with that of the *N*-linked sites, suggesting that *O*-linked glycosylation is a limited local modification that does not appear to affect the overall glycan density.

Regarding *O*-linked glycosylation, single glycosylation forms were observed at the T32, S134, S135, T147, T156, T162, T194, S197, S207, T233, S237, S249, and S255 residues in HA1-Mut^+^ (Additional file 3), and at the T18, T20, S73, T126, S135, T147, S158, S197, S207, T233, and S260 residues in HA1-Mut^S^ (Additional file 5). Notably, T20 exhibited the highest *O*-linked glycosylation efficiency in both HA1-Mut^S^ and HA1-Mut^+^, accounting for 86.56% and 70.44% of total *O*-linked glycosylation, respectively (Additional file 6). However, the glycan at this site in HA1-Mut^S^ predominantly consists of Hex_2_ (Additional file 8), whereas in HA1-Mut^+^, it is predominantly Hex_4_ with a larger molecular weight (Additional file 8B). Among all the *O*-linked glycosylation sites in HA1-Mut^+^, the second highest efficiency was observed at T36, which primarily shows the Hex_1_ glycan type (Additional file 8D), matching the glycosylation form observed at this site in HA1-Mut^S^ (Additional file 8C), although the glycosylation efficiency is much lower in HA1-Mut^S^. The third highest *O*-linked glycosylation efficiency was observed at the T162 site, where HA1-Mut^S^ exhibited a slightly higher glycosylation efficiency than HA1-Mut^+^, with a difference of only 1.59%, and the glycan types were consistent (Additional file 8E, F; Additional files 6, 7). Both proteins share four fully overlapping *O*-linked glycosylation sites (S/T135, 147, 197, and 207), which exhibit the same glycan structure (predicted glycosylation forms for S135 and S207 are shown in Additional file 8G–J). The glycosylation efficiency at these four sites is relatively low, but HA1-Mut^+^ shows lower glycosylation levels at these positions. Although both proteins exhibit a single glycan type at the T233 site, their glycan compositions differ (HA1-Mut^S^ with Hex_12_ and HA1-Mut^+^ with Hex_9_). Additionally, HA1-Mut^+^ contains more complex *O*-linked glycans, such as Hex_2_Phospho_1_ at the S165, S176, and T183 sites and Hex_2_Sulfo_1_ at the T116 site (Additional file 3). HA1-Mut^S^ also contains Hex_2_Phospho_1_ at the T32, S165, S176, and S198 sites and Hex_2_Sulfo_1_ at the S176 and T235 sites (Additional file 5). In comparison, the *O*-linked glycosylation efficiency at T20, T36, T162, S135, and S207 in HA1-Mut^S^ is lower than that in HA1-Mut^+^ (Additional file 8K, L). HA1-X33 exhibits even more complex *O*-linked glycosylation, such as Hex_12_ (Additional file 9A), Hex_14_ (Additional file 9B), and Hex_16_ (Additional file 9C) at the T23 site. These results suggest that HA1-Mut^+^ exhibits a more diverse *O*-linked glycosylation pattern, including additional phosphorylation and sulfation glycans, while HA1-Mut^S^ shows a more simplified *O*-linked glycosylation profile.

Collectively, the results of LC–MS/MS analysis confirm that the type as well as abundance of *N*-linked and *O*-linked glycans are different between HA1-Mut^+^ and HA1-Mut^S^. Moreover, HA1-Mut^+^ exhibits a higher degree of glycosylation with a greater proportion of high-mannose type of glycans, while HA1-Mut^S^ contains fewer high-mannose chains and exhibits reduced glycan heterogeneity. These findings demonstrate that the SuperMan5 strains of *K. phaffii* can effectively suppress hypermannosylation, resulting in a more homogeneous, simplified oligomannose profile. While this truncated glycan structure does not replicate the complex *N*-linked glycans typically found on native mammalian-derived HA, it successfully circumvents the highly immunogenic yeast-specific hypermannosylation. This simplification is crucial for reducing steric hindrance and unmasking immunodominant neutralizing epitopes.

### In vitro self-assembly of HA1-Mut^S^ with ferritin and structural characterization of virus-like particles (VLPs)

**Figure 4:**
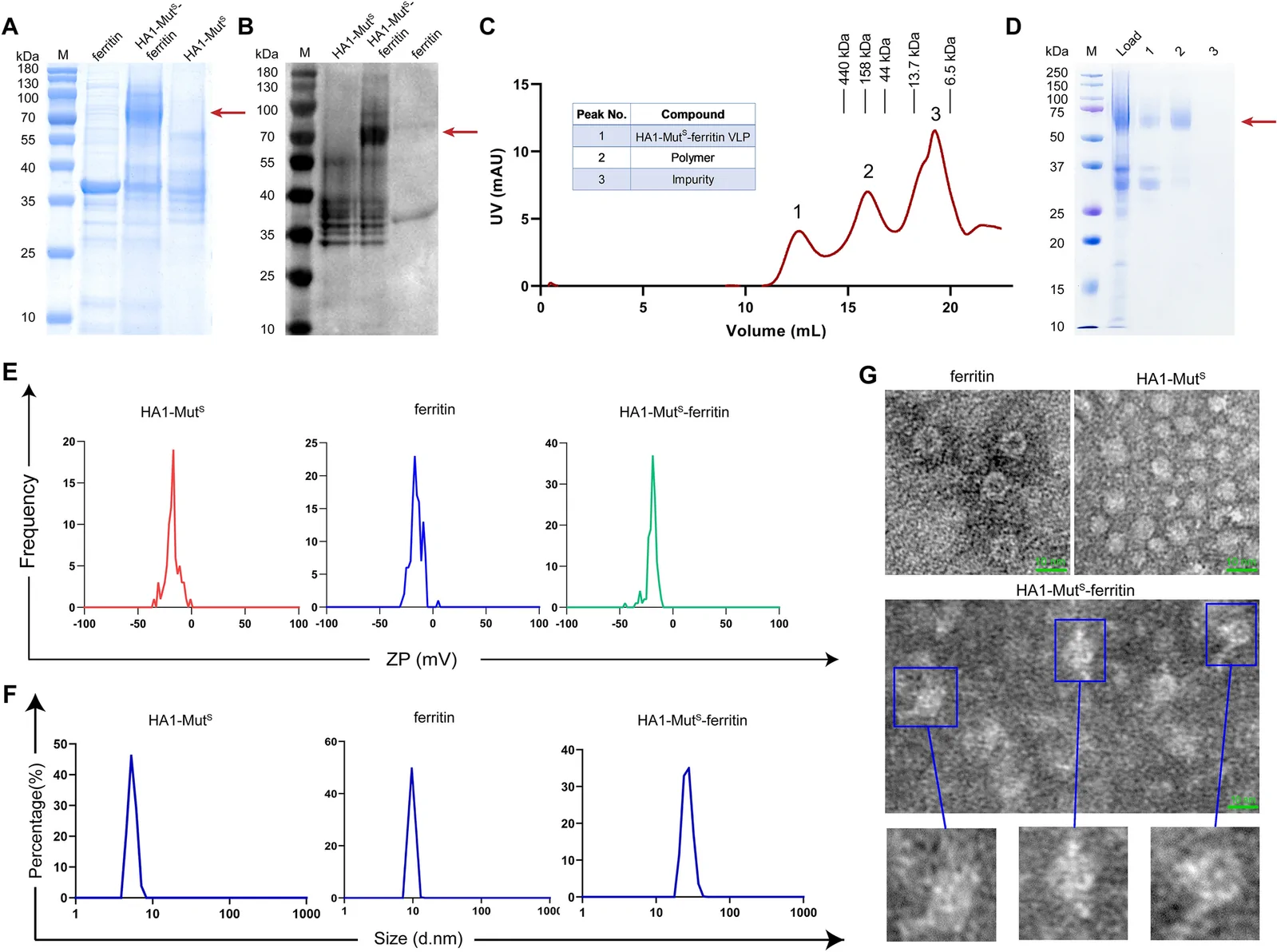
**In vitro self-assembly and structural characterization of HA1-Mut**^**S**^**-ferritin.** **A** Reducing SDS-PAGE of VLPs formed by the binding of HA1-Mut^S^ and ferritin. The disappearance of ferritin in the HA1-Mut^S^-ferritin lane indicates covalent linkage. **B** Western blot of VLPs formed by the binding of HA1-Mut^S^ and ferritin. Ferritin is covalently linked to HA1-Mut^S^, confirming VLP formation. **C**, **D** SEC of the HA1-Mut^S^-ferritin covalent complex on a Superose 6 Increase 10/300 GL column. VLPs were collected at retention peak 1. **E** Zeta potential characterization of HA1-Mut^S^ monomer, ferritin monomer, and VLPs. All 3 exhibit stable, well-dispersed colloidal properties, with the assembled VLPs demonstrating a shift in zeta potential to −20 mV. **F** DLS analysis of HA1-Mut^S^, ferritin, and the assembled HA1-Mut^S^-ferritin VLPs. The hydrodynamic diameters of HA1-Mut^S^ and ferritin were approximately 7 nm and 10 nm, respectively, which increased to ~30 nm upon assembly into nanoparticles. **G** TEM images of unlinked HA1, ferritin, and assembled VLPs. The HA1-Mut^S^-ferritin complex adopts a polyhedral shape, with an overall diameter of approximately 30 nm.

Based on the results of the affinity and glycosylation MS experiments, we selected HA1-Mut^S^ with lower glycosylation levels for the next phase of experimentation. Following the self-assembly of HA1-Mut^S^ and ferritin under in vitro conditions, the products were analyzed via SDS-PAGE and western blot analysis alongside equimolar and equal volume single-component controls for assessing assembly efficiency. As shown in Figure 4A, ferritin (~38 kDa) and HA1-Mut^S^ (30–40 kDa) self-assembled to form the fusion protein HA1-Mut^S^-ferritin with an apparent molecular weight of 68–78 kDa. Notably, the optimal conjugation ratio of ferritin to HA1-Mut^S^ was determined to be 1:3, as this ratio resulted in the most efficient assembly (Additional file 10). Western blot analysis further validated the specificity of the assembly process (Figure 4B). The HA1-Mut^S^-ferritin complex was specifically recognized by His-tagged monoclonal antibody without significant nonspecific bands, confirming the specificity and efficiency of SpyTag/SpyCatcher-mediated covalent coupling. Prior to structural characterization, the HA1-Mut^S^-ferritin assembly was purified and analyzed using SEC. Size-exclusion chromatography revealed three major peaks, with peak 1 corresponding to the molecular weight range of the HA1-Mut^S^-ferritin assembly (Figure 4C). SDS-PAGE analysis confirmed that the size of peak 1 corresponds to the theoretical size of the HA1-Mut^S^-ferritin monomer (Figure 4D). In short, efficient in vitro self-assembly of homogeneous VLP nanoparticles was achieved herein, which were further purified using SEC for downstream structural and functional investigations.

The resulting self-assembled nanoparticles were comprehensively evaluated via SDS-PAGE, Zeta potential, TEM, and DLS analyses. SDS-PAGE analysis revealed that all three fusion proteins exhibited single, distinct bands at approximately 35 kDa under various storage conditions (−20 °C and 4 °C for 14 and 28 days), with no visible degradation products, indicating high purity and excellent storage stability (Additional file 11). An analysis of Zeta potential distribution revealed single and narrow peaks for HA-Mut^S^, ferritin, and HA-Mut^S^-ferritin, indicating good dispersion and high colloidal stability (Figure 4E). The average Zeta potential values for HA-Mut^S^ and ferritin were approximately −10 and −15 mV, respectively, which further shifted to approximately −20 mV for the HA-Mut^S^-ferritin nanoparticles, suggesting significant alterations of surface charge properties during the self-assembly process. The enhanced negative surface charge may result from increased exposure of acidic residues (Asp and Glu) on HA-Mut^S^ after the self-assembly process, the intrinsic negative charge of the ferritin scaffold, and reduced electrostatic shielding due to lower levels of glycosylation. A moderate increase in surface negativity typically enhances electrostatic repulsion, thereby improving colloidal stability. Moreover, in the context of vaccine delivery, negatively charged nanoparticles typically exhibit better biocompatibility and are taken up more efficiently by antigen-presenting cells, which is beneficial for subsequent immune activation. After the self-assembly process, the HA1-Mut^S^-ferritin complexes appeared as well-defined, polyhedral spherical nanoparticles with an overall diameter of approximately 30 nm. Distinct spike-like protrusions corresponding to HA1-Mut^S^ were evenly distributed on the surface of ferritin, indicating a highly ordered assembly. This structural organization was further validated via DLS analysis, which revealed hydrodynamic diameters of ~10 and ~7 nm for ferritin and HA1-Mut^S^, respectively. Upon nanoparticle formation, the hydrodynamic diameter increased to ~30 nm (Figure 4F). TEM imaging further elucidated the spatial architecture of the assembled complexes, with ferritin displaying a typical hollow cage-like morphology and an average diameter of approximately 10 nm, consistent with its native conformation (Figure 4G). These findings demonstrate that HA1-Mut^S^ and ferritin can spontaneously self-assemble into stable and reproducible nanostructures under in vitro conditions. Taken together, the results of Zeta potential, TEM, and DLS analyses indicate that HA1-Mut^S^ spontaneously and consistently assembles with ferritin to form uniform and stable nanoparticle complexes.

### HA1-Mut^S^-ferritin-CpG vaccine elicits an antibody-mediated immune response

**Figure 5:**
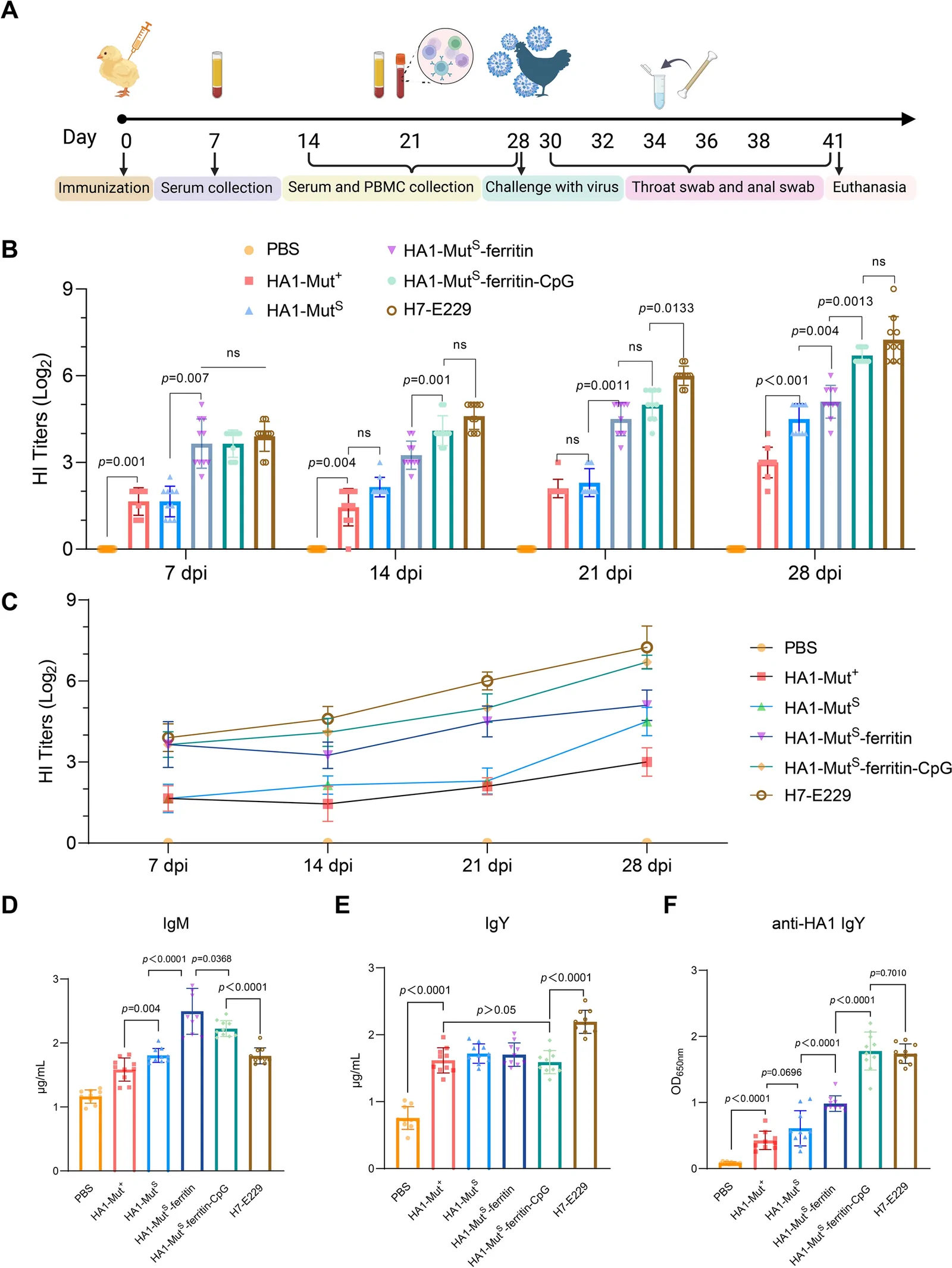
**Induction of humoral immune response by HA1-Mut**^**S**^**-ferritin vaccine in SPF chickens.** **A** Schematic diagram of immune response and roster program in animal studies, created using BioRender. **B**, **C** Serum-specific antibody titers against H7 E229 avian influenza virus strain in SPF chickens, measured by HI assay at 7, 14, 21, and 28 dpi. Titers of IgM (**D**), IgY (**E**), and specific IgY antibodies **F** in sera collected from the blank group, HA1-Mut^+^, HA1-Mut^S^, HA1-Mut^S^-ferritin, HA1-Mut^S^-ferritin-CpG, and E229 groups at 28 dpi. The HA1-Mut^S^-ferritin-CpG vaccine elicited high-titer, sustained HA1-specific antibody responses at 28 dpi that were comparable to those induced by the inactivated H7–E229 vaccine, with the rapid IgM-to-IgY isotype switch indicating efficient initiation of primary humoral immunity and maturation. Data are presented as mean ± SD for each group. ^*^p < 0.05; ^**^p < 0.01; ^***^p < 0.001; ns, not significant.

To systematically evaluate the humoral immunogenicity of the HA1-Mut^S^-ferritin-CpG vaccine, specific pathogen-free (SPF) chickens were immunized with the vaccine, while equivalent amounts of the proteins HA1-Mut^S^ and HA1-Mut^+^ as well as PBS were employed for the control groups (Figure 5A). Hemagglutination inhibition (HI) assays were performed using four HA units of inactivated H7–E229 virus as the antigen to assess the serum levels of virus-specific antibodies (Figure 5B). The HA1-Mut^S^-ferritin-CpG group elicited a relatively high HI titer (mean log_2_ value of 3.65) on 7 dpi, comparable to that of the groups immunized with HA1-Mut^S^-ferritin and inactivated H7–E229 virus (log_2_ values of 3.65 and 3.9, respectively; *p* > 0.05) and significantly higher than that of the HA1-Mut^S^ group (log_2_ value of 1.65; *p* = 0.007). By 14 dpi, increased HI titers were obtained for all the groups, with the HA1-Mut^S^-ferritin-CpG group maintaining a superior titer (log_2_ value of 4.1) that was significantly higher than that of the HA1-Mut^S^-ferritin group (log_2_ value of 3.25; *p* = 0.001), while remaining comparable to that of the H7–E229 group (log_2_ value of 4.6; *p* > 0.05). By contrast, the HI titer of the HA1-Mut^+^ group remained low (log_2_ value of 1.45). The titer of the HA1-Mut^S^-ferritin-CpG group further increased to 5.0 (log_2_) on 21 dpi, which was slightly higher than that of the HA1-Mut^S^-ferritin group (log_2_ value of 4.5; *p* > 0.05). The H7–E229 group maintained the highest titer (log_2_ value of 6.0), which was significantly higher than that of the HA1-MutS-ferritin-CpG group (*p* = 0.0133). By contrast, the HI titer of the HA1-Mut^S^ and HA1-Mut^+^ (log_2_ values of 2.3 and 2.1, respectively) groups remained significantly lower than H7–E229 (*p* = 0.0011). By 28 dpi, the HI titers (log_2_ values) of the H7–E229, HA1-Mut^S^-ferritin-CpG, HA1-Mut^S^-ferritin, HA1-Mut^S^, and HA1-Mut^+^ groups increased to 7.25, 6.7, 5.1, 4.5, and 3.0, respectively; however, statistically significant difference was not observed between the HA1-Mut^S^-ferritin-CpG and H7–E229 groups (*p* > 0.05). No antibodies were detected in the PBS control group throughout the study. Overall, all the immunized groups exhibited gradually increasing HI titers, with the H7–E229 inactivated virus vaccine eliciting the most rapid and strongest response (Figure 5C). The antibody levels attained in the HA1-Mut^S^-ferritin-CpG group were comparable to that of the H7–E229 group and significantly higher than those of the HA1-Mut^S^-ferritin and HA1-Mut^S^ groups; the HA1-Mut^+^ group consistently showed the lowest response. These results indicate that multivalent antigen presentation via the ferritin-based nanoparticles, in conjunction with the use of the adjuvant CpG, synergistically enhances the humoral immunogenicity of the antigen HA1-Mut^S^.

To further assess the immunogenicity of the subunit vaccines, the titers of HA1-specific antibodies in chicken sera were estimated using ELISA. An analysis of antibody isotypes showed that the HA1-Mut^S^-ferritin-CpG group induced robust HA1-specific immunoglobulin M (IgM) responses at the early time points (Figure 5D), which gradually switched to an IgY-mediated response; this finding indicates the rapid initiation of primary humoral immunity, followed by B-cell affinity maturation and cascade amplification of the immune response (Figure 5E). On 28 dpi, the HA1-Mut^S^-ferritin-CpG and the H7–E229 group exhibited the highest titers of HA1-specific antibodies, significantly exceeding those of the control groups (Figure 5F), indicating that the adjuvant CpG substantially enhanced the humoral immune response. These results demonstrate that the HA1-Mut^S^-ferritin-CpG vaccine induces humoral immune response comparable to that induced by the inactivated H7–E229 virus vaccine, generating high-titer, antigen-specific antibodies in a rapid and sustained manner. Furthermore, the antigen HA1-Mut^S^-ferritin elicited higher antibody titers than HA1-Mut^S^, demonstrating that ferritin nanoparticles significantly improved the immunogenicity of the HA1 antigen.

### HA1-Mut^S^-ferritin-CpG vaccine elicits a cellular immune response

**Figure 6:**
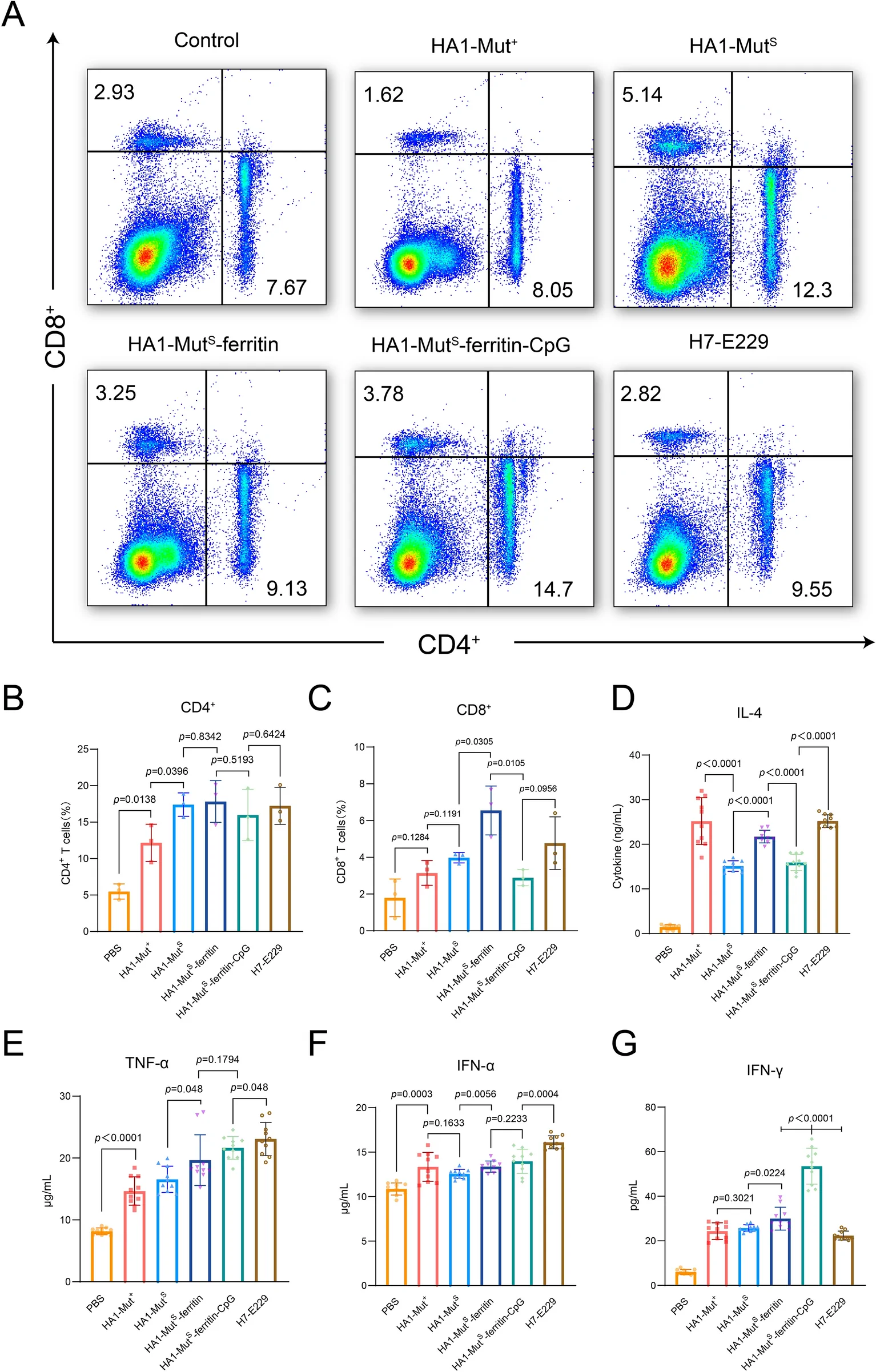
**Cellular immune response in peripheral blood mononuclear cells from chickens immunized with blank control, HA1-Mut**^**+**^**, HA1-Mut**^**S**^**, HA1-Mut**^**S**^**-ferritin, HA1-Mut**^**S**^**-ferritin-CpG, and E229 vaccines.** **A**–**C** Proportions of CD4^+^ and CD8^+^ T cells in PBMCs of chickens immunized with PBS or different antigens at 28 dpi, as analyzed by flow cytometry. The HA1-Mut^S^-ferritin and HA1-Mut^S^ groups exhibited the highest proliferation rates of CD4^+^ T cells and expansion rates of CD8^+^ T cells. PBMCs isolated from immunized chickens at 28 dpi were stimulated in vitro with purified HA1 protein, and the levels of IL-4 **D**, TNF-α **E**, IFN-α **F**, and IFN-γ **G** in the culture supernatants were measured by ELISA. The HA1-Mut^S^-ferritin-CpG vaccine induced a strong cellular immune response, with particularly significant effects on IFN-γ induction. Data are presented as mean ± SD for each group. ^*^p < 0.05; ^**^p < 0.01; ^***^p < 0.001; ns, not significant.

The cellular immune response elicited by the subunit vaccine was subsequently evaluated via flow cytometry analysis of the proportions of cluster of differentiation 4-positive (CD4^+^) and cluster of differentiation 8-positive (CD8^+)^ T-lymphocytes in the peripheral blood of immunized SPF chickens (Figure 6A). At three weeks post-immunization, all the vaccinated groups exhibited significant increases in both CD4^+^ and CD8^+^ T cell populations compared with those of the PBS control (Figure 6B, C). Among these, the HA1-Mut^S^-ferritin and HA1-Mut^S^ groups exhibited the highest rates of CD4^+^ and CD8^+^ T cell proliferation expansion, respectively. Subsequently, cytokine levels were measured in the culture supernatants of PBMCs stimulated with the HA1-Mut^S^ antigen for 24 h. The secretion of interleukin-4 (IL-4) was higher in the HA1-Mut^S^-ferritin group compared with that in the HA1-Mut^S^-ferritin-CpG group, but still significantly lower than that observed in the inactivated H7–E229 virus vaccine group (Figure 6D; *p* < 0.0001). The H7–E229 group exhibited the highest levels of TNF-α and IFN-α, followed by the HA1-Mut^S^-ferritin-CpG group, while the HA1-MutS-ferritin group recorded the lowest levels among the three groups (Figure 6E, F; *p* < 0.05). Notably, significantly higher levels of IFN-γ were induced in the HA1-Mut^S^-ferritin-CpG group compared with that in the HA1-Mut^S^-ferritin or H7–E229 groups (Figure 6G; *p* < 0.0001). These findings demonstrate that the HA1-Mut^S^-ferritin-CpG vaccine elicits robust cellular immune responses, with a particularly strong effect on IFN-γ induction, surpassing the control vaccine lacking CpG.

### Protective efficacy of HA1-Mut^S^-ferritin-CpG vaccine

**Figure 7:**
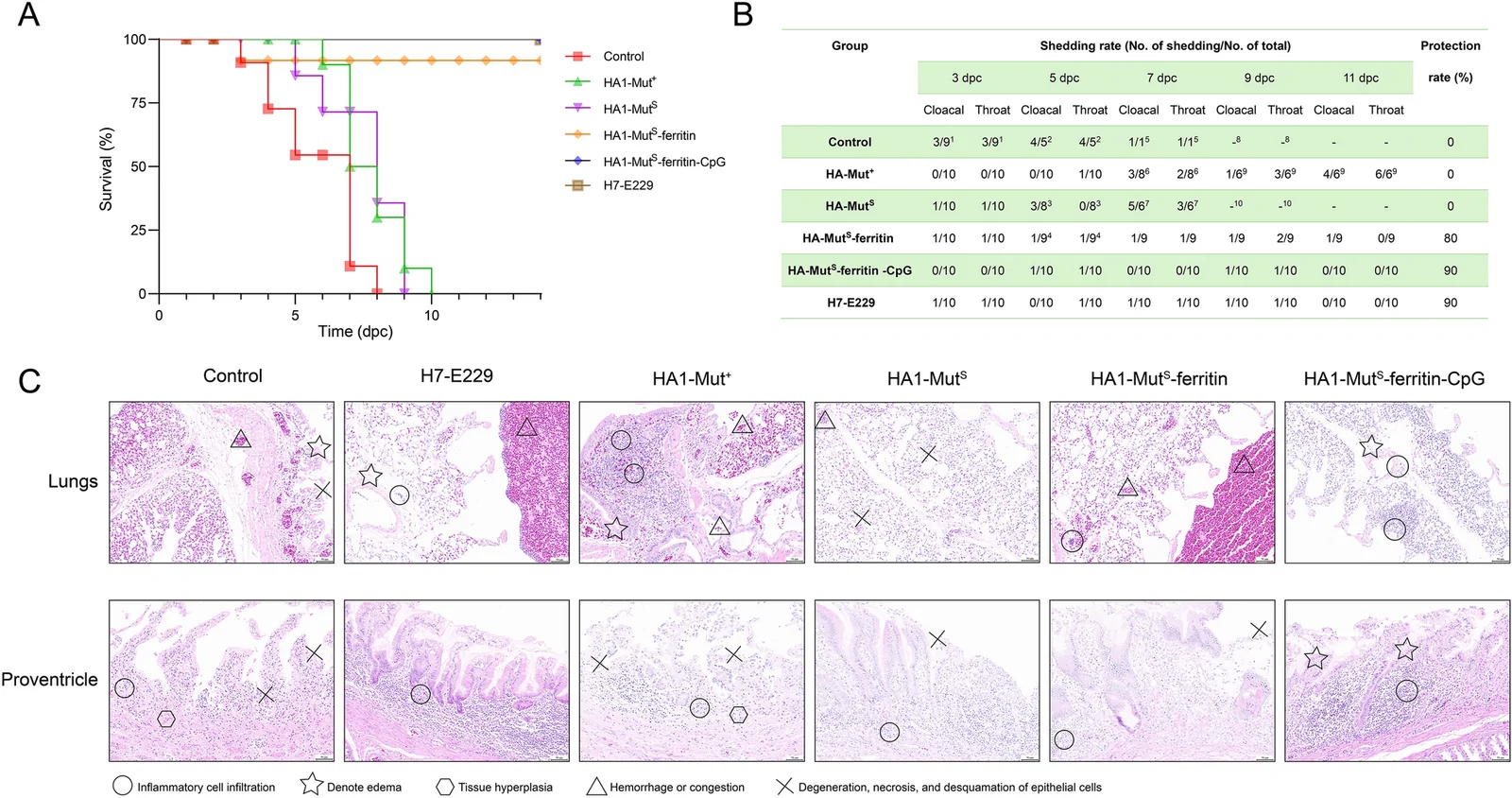
**Protective effects of HA1-Mut**^**+**^**, HA1-Mut**^**S**^**, HA1-Mut**^**S**^**-ferritin, HA1-Mut**^**S**^**-ferritin-CpG, E229 inactivated vaccine, and control vaccine against H7 subtype E229 strain challenge.** At 3, 5, 7, 9, and 11 dpc, cloacal and throat swabs were collected from chickens to track viral shedding via chicken embryo neutralization assay. At 7 dpc, lung and proventriculus samples were collected from chickens immunized with different antigens. **A** Survival rates of immunized groups within 14 dpc following viral challenge. **B** Viral shedding as detected by chicken embryo neutralization assay within 11 dpc. HA1-Mut^S^-ferritin-CpG and E229 inactivated vaccine both demonstrated a 90% shedding rate. **C** Histopathological analysis of lung and proventriculus tissues to assess lesion development (bar = 50 μm). The HA1-Mut^S^-ferritin, HA1-Mut^S^-ferritin-CpG, and H7–E229 groups showed only mild lesions, limited to occasional light edema or inflammatory cell infiltration.

At 28 dpi, the chickens were challenged with avian influenza virus to assess the protective efficacy of the vaccine. The chickens of the PBS group developed clinical symptoms (ruffled feathers and comb cyanosis) within 1–2 dpc with the AIV; mortality was observed from 3 dpc and reached 100% by 8 dpc (Figure 7A). The HA1-Mut^S^ and HA1-Mut^+^ groups showed delayed disease onset (by 9 dpc), but ultimately displayed 100% mortality (by 14 dpc). By contrast, only one chicken of the HA1-Mut^S^-ferritin group was dead by 3 dpc, and all the other birds survived. Notably, clinical symptoms or mortality were not observed among the birds of the HA1-Mut^S^-ferritin-CpG or inactivated H7–E229 groups throughout the observation period. Consistent with survival outcomes, viral shedding was detected from 3 dpc in the PBS, HA1-Mut^S^, HA1-Mut^S^-ferritin, and H7–E229 groups and from 5 dpc in the HA1-Mut^+^ group (Figure 7B). Complete viral clearing was observed in the H7–E229 group by 11 dpc. Viral shedding was transiently observed in only one chicken of the HA1-Mut^S^-ferritin-CpG group on 5 dpc, which was completely cleared by 11 dpc. Observation of the organs revealed significant hemorrhaging in the trachea, heart, liver, proventriculus, gizzard, spleen, and lungs in the vontrol, HA1-Mut^+^, and HA1-Mut^S^ groups (Additional file 12). In contrast, no noticeable hemorrhaging or ulceration was observed in the H7–E229, HA1-Mut^S^-ferritin, and HA1-Mut^S^-ferritin-CpG groups. Histopathology analysis revealed severe lesions in PBS-treated birds, including hemorrhage and necrosis in the respiratory and digestive tissues (Figure 7C), while the control and HA1-Mut^+^ groups showed more pronounced lesions in the liver (Additional file 13), gizzard (Additional file 14), and spleen (Additional file 15), with milder changes observed in the other groups. Similarly, the HA1-Mut^+^ and HA1-Mut^S^ groups showed moderate-to-severe pulmonary and proventricular injury. By contrast, the HA1-Mut^S^-ferritin, HA1-Mut^S^-ferritin-CpG, and H7–E229 groups exhibited minimal lesions, limited to occasional mild edema or inflammatory cell infiltration. Taken together, these data demonstrate that HA1-Mut^S^-ferritin-CpG confers strong protection against viral challenge, effectively preventing clinical disease, mortality, viral shedding, and tissue damage, with a protective profile comparable to that of the inactivated H7–E229 virus vaccine.

In conclusion, the current study validates the feasibility and advantages of yeast-based expression systems for veterinary vaccine production, including eukaryotic post-translational modifications, proper protein folding, and epitope preservation, enabling low-cost, high-efficiency, and animal-free antigen production. Yeast-based recombinant protein platforms provide a safe and scalable route for developing vaccines against avian influenza and other major animal infectious diseases, with broad translational potential.

## Discussion

Similar to mammalian cells, *K. phaffii* has the capacity to support post-translational modifications of expressed proteins; however, high-mannose glycans are frequently added to the expressed proteins, resulting in hyper glycosylation [32]. Moderate glycosylation can enhance the stability, modulate biological activity, and prolong serum half-life of the expressed proteins; however, excessive glycosylation may interfere with proper protein folding and function [33, 34]. The number and linkage patterns of high-mannose glycans depend on the yeast strain and culture conditions, leading to concerns about reproducibility and quality control of yeast-expressed bioproducts [35].

*N*-linked and *O*-linked glycosylation represent the two major types of glycosylation in animals and fungi [36]. In eukaryotic cells, *N*-linked glycans are attached to the amide group of asparagine residues within the consensus sequence Asn-X-Ser/Thr, where X cannot be proline [37]. *O*-linked glycosylation in *K. phaffii* primarily involves short linear chains of α-1, 2-linked mannose residues, which are connected with terminal β-1, 2-linked or phosphorylated mannose residues [38]. Although *N*-linked glycans in *K. phaffii* are structurally different from those in mammalian cells, extensive studies have demonstrated that yeast strains can be engineered for humanized patterns of *N*-linked glycosylation. For example, Jacobs et al. converted Man_8_GlcNAc_2_ to Man_5_GlcNAc_2_ to generate mannose receptor ligands that serve as precursors for human pathways of* N*-linked glycosylation [39, 40]. While fully humanized *N*-linked glycosylation pathways can produce authentic complex glycans, such extensive genetic modifications often compromise cellular fitness and significantly reduce secretory yields [41]. Furthermore, we opted against employing site-directed mutagenesis to directly eliminate native *O*-linked or *N*-linked glycosylation sites. Previous studies have demonstrated that native glycosylation sequons and their associated glycans are intricately linked to HA intracellular folding and the preservation of structural integrity [42]. Therefore, prioritizing the Mut^S^ bioprocess strategy utilizing the *OCH1*-knockout SuperMan5 strain represents a favorable balance for veterinary applications. It effectively truncates the bulky yeast hypermannan shield without sacrificing high-yield production or antigen conformation. In the current study, HA1-Mut^S^ and HA1-Mut^+^ were expressed in the *OCH1*-knockout SuperMan5 and Mut^+^ strains, respectively, of *K. phaffii*. LC–MS/MS analysis revealed differences in glycan composition between the two proteins, with HA1-Mut^+^ containing a higher proportion of *N*-linked high-mannose glycans compared with HA1-Mut^S^, suggestive of an overall higher extent of glycosylation of the former. SDS-PAGE and western blot analyses demonstrated that HA1-Mut^S^ migrated as a uniform band, whereas HA1-Mut^+^ appeared diffuse and poorly resolved (Figure 2B, C), likely due to heterogeneous glycan structures, which is consistent with the results of MS analysis. These findings confirm that the SuperMan5 strain of *K. phaffii* with *OCH1* knockout can be used for producing HA1-Mut^S^ with moderate glycosylation, which maintains the uniformity and functional integrity of the expressed protein.

The excessive glycosylation of HA1-Mut^+^ likely altered its charge, size, and solubility, thereby affecting its electrophoretic mobility and detection potential via western blot analysis. Moreover, glycosylation can modulate antibody binding affinity, potentially reducing assay sensitivity and specificity [43]. Consistent with these observations, in vivo experiments showed that HA1-Mut^+^ elicited weaker immune responses than HA1-Mut^S^, supporting the notion that moderate glycosylation enhances the immunogenicity of antigens [44]. By contrast, excessive glycosylation may mask immunodominant epitopes, thereby reducing vaccine efficacy. Despite these insights, the specific effects of glycan type and structure as well as site-specific modification on HA1 immunogenicity remain incompletely understood and warrant further investigation. The regulation of *O*-linked glycosylation in *K. phaffii* is more challenging, as this modification may occur at positions not present in native proteins [45, 46]. For instance, Dai et al. identified two threonine residues at the C-terminus of recombinant human platelet-derived growth factor–BB as major sites for *O*-linked glycosylation in the yeast-expressed protein [47], while Gong et al. mapped specific sites for*O*-linked glycosylation in *Pichia*-derived recombinant human granulocyte colony-stimulating factor based on proteolytic and chemical cleavage [48]. The site and sequence specificity of *O*-linked glycosylation vary among proteins, and studies on this modification are often protein specific. Radoman et al. analyzed 16 different proteins via LC/electrospray ionization (ESI)-MS and found that slower-growing yeast strains produced proteins with a higher extent of glycosylation [37]. In the current study, HA1-Mut^+^ contained a greater diversity of *O*-linked glycans than HA1-Mut^S^ (Additional file 3), which can potentially lead to epitope masking and can account for its reduced in vivo immunogenicity. The mechanisms underlying these phenomena warrant further exploration. In addition to optimizing glycosylation, the structural conformation and spatial presentation of the antigen are critical determinants of vaccine efficacy. The purified recombinant HA1 subunit in our study is primarily monomeric in solution. Typically, the expression of the full-length HA extracellular domain requires its native stem region (HA2) or an exogenous trimerization motif (e.g., T4 foldon) to form stable trimers [49]. However, our rational vaccine design intentionally utilized the monomeric HA1 subunit for two critical reasons. First, expressing the monomeric HA1 helps mitigate the folding and aggregation issues often associated with the hydrophobic stem regions in yeast expression systems, a strategy that contributed to achieving the high yields (1.21 g/L) necessary for cost-effective veterinary applications. Second, restricting the antigen primarily to HA1 may enhance the exposure of the receptor-binding domain, which helps to direct the host immune response predominantly toward major neutralizing epitopes while reducing off-target responses. While monomeric antigens often exhibit weaker immunogenicity in isolation, our use of the SpyTag/SpyCatcher system to covalently array these monomers onto a 24-mer ferritin nanocage creates an effective multivalent display. As demonstrated in previous studies on nanoparticle vaccines for both influenza and SARS-CoV-2, this multivalent presentation can compensate for the lack of native trimerization by facilitating B-cell receptor cross-linking, which likely contributed to the robust immune responses observed in our study [50–52]. Furthermore, this specific SpyTag/SpyCatcher bioconjugation is designed to promote a uniform and directional display of the HA1 antigen on the ferritin nanocage [53]. Because the isopeptide bond forms specifically at the designated fusion termini, the assembly pattern is highly consistent, firmly anchoring the base of the HA1 monomer to the core. While this specific orientation inevitably masks some basal epitopes, it provides a crucial immunological advantage by structurally forcing the outward presentation of the receptor binding domain (RBD) and major neutralizing epitopes [54]. Consistent with this, our structural modeling (Figure 1B) suggests a relatively loose spatial arrangement where these critical sites remain highly exposed and unmasked. Crucially, the efficiency of this precise covalent assembly is heavily dependent on the antigen’s glycosylation profile. Bulky glycans are known to introduce severe steric hindrance that can physically block specific protein–protein interactions [12]. Therefore, the truncated oligomannose profile generated by our engineered Mut^S^ strain, combined with this spatial separation, is not only essential for exposing these neutralizing epitopes to the immune system but also fundamentally necessary for minimizing steric shielding to ensure the successful and highly efficient assembly of the VLPs.

CpG oligodeoxynucleotides (ODNs) are synthetic DNA sequences that contain unmethylated cytosine-phosphate-guanine motifs and mimic microbial DNA. CpG ODNs activate the immune system via toll-like receptor 9 (TLR9), enhancing vaccine immunogenicity [55]. CpG is widely used as an adjuvant in vaccine formulations. The currently available influenza vaccines are typically effective against highly homologous strains but offer limited cross-protection. CpG ODNs represent a promising class of adjuvants that have been applied successfully in various vaccine formulations. For example, Shirai et al. demonstrated that lipid nanoparticles delivering CpG ODNs enhanced the protective efficacy of split influenza vaccines in mice [56]. A combination of CpG with an aluminum adjuvant increased the titers of neutralizing antibodies against severe acute respiratory syndrome coronavirus 2 in aged mice by 80-fold, providing protection against viral challenge [57].

In the current study, CpG was used as an adjuvant for a vaccine formulation containing HA1-Mut^S^ and ferritin VLPs; this vaccine was then subcutaneously administered to 2-week-old SPF chickens. Serum and peripheral blood lymphocytes were collected weekly to assess vaccine induced immunity. The inclusion of CpG significantly enhanced humoral responses, with mean serum antibody titers (log_2_ values) of 4.1 by week 2, which steadily increased to 5.0 and 6.7 by weeks 3 and 4, respectively. This titer was significantly higher than that of the HA1-Mut^S^-ferritin group without CpG, confirming the effect of the adjuvant on humoral immune responses (Figure 5B, C). Flow cytometry analysis of T cell responses revealed that CpG had a relatively modest effect on cell-mediated immunity. CD4^+^ and CD8^+^ T cell activation and proliferation were slightly higher with HA1-Mut^S^-ferritin-CpG than with HA1-Mut^S^-ferritin (Figure 6A). This modest quantitative enhancement in T cell proliferation is likely due to a “ceiling effect", as the multivalent VLP presentation combined with the robust SEPPIC ISA 71VG oil emulsion already elicited an exceptionally strong baseline cellular response. Nevertheless, CpG critically modulated the qualitative aspects of the immune response by driving robust IFN-γ secretion (Th1 polarization) and accelerating the IgM-to-IgY isotype switch. Regarding the immune correlates of protection (CoP), our data suggest a complementary protective profile. As the widely accepted primary humoral CoP, our vaccine elicited peak HI titers (6.7 log_2_) that were comparable to the conventional H7–E229 vaccine (7.25 log_2_), with both groups achieving 100% survival. Additionally, our nanoparticle formulation induced elevated levels of IFN-γ secretion compared with the conventional counterpart (Figure 6G), which may serve as a potential secondary cellular CoP. The relevance of these combined immune correlates is supported by the in vivo chicken embryo neutralization assay (Figure 7B). This assay demonstrated that the immune profile elicited by the nanoparticle vaccine effectively curtailed viral shedding, achieving virological clearance comparable to the conventional vaccine. Furthermore, adverse effects were not observed in immunized chickens, which exhibited normal behavior and diet. Overall, CpG is an immunostimulatory adjuvant with broad potential applications in vaccine development, cancer immunotherapy, anti-infective therapy, immunomodulation, and next-generation vaccines. Ongoing mechanistic studies and technological advances are expected to further expand its potential applications.

Looking toward future industrial application, the engineered *K. phaffii* SuperMan5 strain is designed to restrict hypermannosylation by knocking out the *OCH1* gene, providing a more consistent glycosylation baseline. However, scaling up fermentation can introduce variability in culture conditions (e.g., dissolved oxygen and localized methanol concentrations in large bioreactors), which may subtly affect the final glycosylation profile. Therefore, careful bioprocess optimization, particularly regarding the methanol feeding strategy, will be important to maintain lot-to-lot consistency during potential commercial manufacturing. Furthermore, a known limitation of conventional inactivated vaccines is their vulnerability to the antigenic drift of circulating influenza strains, as adapting new viruses for egg-based production can be time-consuming. Our modular platform offers a potential strategy to address this challenge. The SpyTag/SpyCatcher system allows the ferritin nanocage to act as a versatile, premanufactured scaffold. Upon the emergence of a new viral clade, the updated HA1 sequence could be cloned and secreted by the yeast system, facilitating the formulation of matched vaccines without relying on viral adaptation. Additionally, this modular platform is adaptable to multivalent vaccine designs. By coassembling HA1 antigens from diverse circulating clades or subclades onto a single ferritin scaffold (generating mosaic nanoparticles), or by formulating cocktails of varied VLPs, future iterations of this platform could help broaden cross-reactive immunity to counter antigenic drift [51, 52]. Although a formal cost-of-goods (COGs) assessment remains for future pilot-scale studies, a preliminary projection based on our experimental data suggests potential economic advantages over conventional inactivated vaccines. The Mut^S^ strain achieved a secretion yield of 1.21 g/L. Assuming a conservative downstream purification recovery of 50%, one liter of fermentation broth yields approximately 0.6 g of pure HA1 antigen. Given our in vivo immunization dose of 60 µg per bird, a single liter of yeast culture can theoretically supply up to 10000 vaccine doses. This highlights the potential economic viability of the platform, aligning with recent studies that demonstrate the scalability and cost efficiency of *K. phaffii* manufacturing at the pilot scale [16]. Additionally, this recombinant approach circumvents the reliance on SPF embryonated chicken eggs, which are associated with supply chain complexities and higher costs [14]. Finally, while producing conventional inactivated vaccines for highly pathogenic strains such as H7 requires BSL-3 manufacturing containment, our recombinant platform utilizes noninfectious components. This allows it to operate under standard industrial conditions (BSL-1), potentially reducing facility overhead, energy consumption, and biosafety compliance costs.

## Supplementary information

**Additional file 1.** **SDS-PAGE identification of the recombinant protein HA1-X33 expressed by the** ***K. phaffii*** **expression system.** SDS-PAGE image showing the expression of the recombinant protein HA1-X33 in the *K. phaffii* expression system, exhibiting diffuse bands between 70 and 100 kDa.

**Additional File 2**. **Analysis of** ***N*-linked glycoforms and glycosylation sites of HA1-Mut**^**+**^**.** Table detailing the *N*-linked glycoforms and glycosylation sites of HA1-Mut^+^, including the position, peptide sequence, glycan structure, modification type, molecular masses (observed and calculated), mass error, identification score, and relative intensity.

**Additional File 3**. **Analysis of *O*-linked glycoforms and glycosylation sites of HA1-Mut**^***+***^**.** Table detailing the *O*-linked glycoforms and glycosylation sites of HA1-Mut^+^, including the position, peptide sequence, glycan structure, modification type, molecular masses (observed and calculated), mass error, identification score, and relative intensity.

**Additional File 4.** **Analysis of** ***N*-linked glycoforms and glycosylation sites of HA1-Mut**^**S**^**.** Table detailing the *N*-linked glycoforms and glycosylation sites of HA1-Mut^S^, including the position, peptide sequence, glycan structure, modification type, molecular masses (observed and calculated), mass error, identification score, and relative intensity.

**Additional File 5**. **Analysis of** ***O*-linked glycoforms and glycosylation sites of HA1-Mut**^**S**^. Table detailing the *O*-linked glycoforms and glycosylation sites of HA1-Mut^S^, including the position, peptide sequence, glycan structure, modification type, molecular masses (observed and calculated), mass error, identification score, and relative intensity.

**Additional File 6.** **Site efficiency analysis of** ***O*-linked and *N*-linked glycosylation in HA1-Mut**^**+**^. Table detailing the site efficiency analysis of *O*-linked and *N*-linked glycosylation in HA1-Mut^+^, including the position, type, sum intensity, and glycosylation efficiency percentage.

**Additional File 7**.**Site efficiency analysis of *O*-linked and *N*-linked glycosylation in HA1-Mut**^**S**^. Table detailing the site efficiency analysis of *O*-linked and *N*-linked glycosylation in HA1-Mut^S^, including the position, type, sum intensity, and glycosylation efficiency percentage.

**Additional File 8.** ***O*-linked glycosylation analysis of HA1-Mut**^**S**^ **and HA1-Mut**^**+**^. Mass spectrometric analysis of *O*-linked glycosylation at T20, T36, T162, S135, and S207 in HA1-Mut^S^ and HA1-Mut^+^, along with a comparison of glycosylation efficiencies demonstrating that HA1-Mut^S^ exhibits a simplified *O*-linked glycosylation profile compared with HA1-Mut^+^.

**Additional File 9.** **Mass spectrometric identification of *O*-linked glycosylation at the T23 site of HA1-X33.** Mass spectrometric identification of *O*-linked glycosylation at the T23 site of HA1-X33, demonstrating that HA1-X33 exhibits more complex *O*-linked glycosylations.

**Additional File 10.** **SDS-PAGE analysis to determine the optimal ratio for in vitro assembly of the HA1-Mut**^**S**^**-ferritin fusion protein.** SDS-PAGE analysis evaluating the in vitro assembly of the HA1-Mut^S^-ferritin fusion protein at different ratios, demonstrating that a 1:3 ratio of ferritin to HA1 is optimal as indicated by the complete disappearance of the ferritin band.

**Additional File 11.** **SDS-PAGE analysis of the storage stability of HA1-Mut**^**+**^**-ferritin, HA1-X33-ferritin, and HA1-Mut**^S^**-ferritin fusion proteins at −20 °C and 4 °C for 14 and 28 days.** SDS-PAGE analysis evaluating the storage stability of HA1-Mut^+^-ferritin, HA1-X33-ferritin, and HA1-Mut^S^-ferritin fusion proteins when stored at −20 °C and 4 °C for durations of 14 and 28 days.

**Additional File 12.** **Organ macroscopic lesions in the H7-E229, Control, HA1-Mut**^**S**^**, HA1-Mut**^**+**^**, HA1-Mut**^**S**^**-ferritin, and HA1-Mut**^**S**^**-ferritin-CpG vaccine groups.** Photographs evaluating macroscopic lesions in the trachea, heart, liver, spleen, gizzard, proventriculus, and lungs at 3 days post-challenge, demonstrating no significant hemorrhaging or erosion in the H7-E229, HA1-Mut^S^-ferritin, and HA1-Mut^S^-ferritin-CpG groups.

**Additional File 13.** **Pathological sections of livers from chickens in each group at 3 dpc.** Histopathological images of liver sections at 3 days post-challenge, demonstrating substantial vascular congestion and focal hepatocyte necrosis in the control and HA1-Mut^+^ groups, whereas the HA1-Mut^S^-ferritin, HA1-Mut^S^-ferritin-CpG, and H7-E229 groups exhibited only mild cellular swelling without other significant abnormalities.

**Additional File 14.** **Pathological sections of gizzards from chickens in each group at 3 dpc**. Histopathological images of gizzard sections at 3 days post-challenge, demonstrating extensive necrosis and shedding of mucosal epithelial cells in the control group, and a notable presence of eosinophilic material in the gastric gland lumens in the HA1-Mut^+^ group, while the other groups showed only minimal eosinophilic material.

**Additional File 15.** **Pathological sections of spleens from chickens in each group at 3 dpc.** Histopathological images of spleen sections at 3 days postchallenge, demonstrating that the control and HA1-Mut^+^ groups showed a higher amount of necrotic cell debris, while the other groups exhibited only mild congestion or swelling.

**Additional File 16.** **Sequences used in this study.** Table listing the amino acid sequences used in this study, including the native and optimized sequences for H7N9 HA1 and ferritin, along with their respective characteristics and accession numbers.

**Additional File 17.** **Primers used in this study.** Table listing the primers used in this study, including the primer names, sequences, and their characteristics.

**Additional File 18.** **Strains and plasmids used in this study.** Table listing the strains and plasmids used in this study, including their characteristics and sources.

## Data Availability

No datasets were generated or analyzed during the current study.
