# Supplementary material for: A glycoengineered yeast-derived HA1-ferritin nanoparticle vaccine confers protective immunity against H7 avian influenza in chickens
Source: Vet Res. 2026 Oct 29;57:190. doi: 10.1186/s13567-026-01867-y (PMC13721490; doi:10.1186/s13567-026-01867-y)
Supplement: Supplementary file 1 [file 13567_2026_1867_MOESM10_ESM.docx]

**Title**: A glycoengineered yeast-derived HA1-ferritin nanoparticle vaccine confers protective immunity against H7 avian influenza in chickens

**Authors**: Yutong Liu, Yunlin Shao, Bo Su, Peiqi Liu, Yifeng Huang, Han Zheng, Siqi Xu, Yishan Zhao, Qianyu Li, Ming Liao, Saixiang Feng

**Additional file 10.** SDS-PAGE analysis to determine the optimal ratio for *in vitro* assembly of the HA1-Mut^S^-ferritin fusion protein. “M” denotes the 180 kDa molecular weight marker. When the ferritin to HA1 ratio was 1:3, the ferritin band completely disappeared, indicating that a 1:3 ratio is the optimal conjugation ratio.
