# Supplementary material for: A glycoengineered yeast-derived HA1-ferritin nanoparticle vaccine confers protective immunity against H7 avian influenza in chickens
Source: Vet Res. 2026 Oct 29;57:190. doi: 10.1186/s13567-026-01867-y (PMC13721490; doi:10.1186/s13567-026-01867-y)
Supplement: Supplementary file 3 [file 13567_2026_1867_MOESM12_ESM.docx]

**Title**: A glycoengineered yeast-derived HA1-ferritin nanoparticle vaccine confers protective immunity against H7 avian influenza in chickens

**Authors**: Yutong Liu, Yunlin Shao, Bo Su, Peiqi Liu, Yifeng Huang, Han Zheng, Siqi Xu, Yishan Zhao, Qianyu Li, Ming Liao, Saixiang Feng

**Additional file 12.** Organ macroscopic lesions in the H7-E229, Control, HA1-Mut^S^, HA1-Mut^+^, HA1-Mut^S^-ferritin, and HA1-Mut^S^-ferritin-CpG vaccine groups. At 3 dpc, chickens were euthanized, and the trachea, heart, liver, spleen, gizzard, proventriculus, and lungs were dissected for evaluation of macroscopic lesions. HA1-Mut^+^, and HA1-Mut^S^ groups across all seven tissues, whereas no significant hemorrhaging or erosion was detected in the H7-E229, HA1-Mut^S^-ferritin, and HA1-Mut^S^-ferritin-CpG groups.
