# Supplementary material for: A glycoengineered yeast-derived HA1-ferritin nanoparticle vaccine confers protective immunity against H7 avian influenza in chickens
Source: Vet Res. 2026 Oct 29;57:190. doi: 10.1186/s13567-026-01867-y (PMC13721490; doi:10.1186/s13567-026-01867-y)
Supplement: Supplementary file 4 [file 13567_2026_1867_MOESM13_ESM.docx]

**Title**: A glycoengineered yeast-derived HA1-ferritin nanoparticle vaccine confers protective immunity against H7 avian influenza in chickens

**Authors**: Yutong Liu, Yunlin Shao, Bo Su, Peiqi Liu, Yifeng Huang, Han Zheng, Siqi Xu, Yishan Zhao, Qianyu Li, Ming Liao, Saixiang Feng

**Additional file 13**. Pathological sections of livers from chickens in each group at 3 dpc. Substantial vascular congestion and focal hepatocyte necrosis in the control and HA1-Mut^+^ groups. The HA1-Mut^S^ group exhibited mild vascular congestion and significant inflammatory cell infiltration. Mild cellular swelling was observed in the HA1-Mut^S^-ferritin, HA1-Mut^S^-ferritin-CpG, and H7-E229 groups, with no other significant abnormalities.
