# Supplementary material for: A glycoengineered yeast-derived HA1-ferritin nanoparticle vaccine confers protective immunity against H7 avian influenza in chickens
Source: Vet Res. 2026 Oct 29;57:190. doi: 10.1186/s13567-026-01867-y (PMC13721490; doi:10.1186/s13567-026-01867-y)
Supplement: Supplementary file 7 [file 13567_2026_1867_MOESM16_ESM.docx]

**Title**: A glycoengineered yeast-derived HA1-ferritin nanoparticle vaccine confers protective immunity against H7 avian influenza in chickens

**Authors**: Yutong Liu, Yunlin Shao, Bo Su, Peiqi Liu, Yifeng Huang, Han Zheng, Siqi Xu, Yishan Zhao, Qianyu Li, Ming Liao, Saixiang Feng

**Additional file 16.** Sequences used in this study.

| Name | Sequence | Characteristic(s) |
| --- | --- | --- |
| A/goose/Guangdong/Q38/2017（H7N9）HA1 | MNTQILVFALIAIIPTNADKICLGHHAVSNGTKVNTLTERGVEVVNATETVERTNTPRICSKGKRTVDLGQCGLLGTITGPPQCDQFLEFSADLIIERREGSDVCYPGKFVNEEALRQILRESGGIDKEPMGFTYNGIRTNGVTSACRRSGSSFYAEMKWLLSNTDNAAFPQMTKSYKNTRESPAIIVWGIHHSVSTAEQTKLYGSGNKLVTVGSSNYQQSFVPSPGARPQVNGQSGRIDFHWLILNPNDTVTFSFNGAFIAPDRASFLRGKSMGIQSGVQVDANCEGDCYHSGGTIISNLPFQNIDSRAVGKCPRYVKQRSLLLATGMKNVPEVPKRKRTARGLFGAIAGFIENGWEGLIDGWYGFRHQNAQGEGTAADYKSTQSAIDQITGKLNRLIAKTNQQFKLIDNEFNEVEKQVGNVINWTRDSITEVWSYNAELLVAMENQHTIDLADSEMDKLYERVKRQLRENAEEDGTGCFEIFHKCDDDCMASIRNNTYDHRKYREEAMQNRIQIDPVKLSSGYKDVILWFSFGASCFILLAIVMGLVFICVKNGNMRCTICI | NCBI accession number: AUD54811.1 |
| Ferritin | MLKPEMIEKLNEQMNLELYSSLLYQQMSAWCSYHTFEGAAAFLRRHAQEEMTHMQRLFDYLTDTGNLPRINTVESPFAEYSSLDELFQETYKHEQLITQKINELAHAAMTNQDYPTFNFLQWYVSEQHEEEKLFKSIIDKLSLAGKSGEGLYFIDKELSTLDTQN | NCBI accession number: WP_000917208.1 |
| H7HA1-optimized for eukaryotic expression | EFMDKICLGHHAVSNGTKVNTLTERGVEVVNATETVERTNIPRICSKGKRTVDLGQCGLLGTITGPPQCDQFLEFSADLIIERREGSDVCYPGKFVNEEALRQILRESGGIDKEPMGFTYNGIRTNGVTSACRRSGSSFYAEMKWLLSNTDNAAFPQMTKSYKNTRESPAIIVWGIHHSVSTAEQTKLYGSGNKLVTVGSSNYQQSFVPSPGARPQVNGQSGRIDFHWLILNPNDTVTFSFNGAFIAPDRASFLRGKSMGIQSGVQVDANCPRYVKQRSLLLATGMKNV | This study |
| Ferritin-optimized for prokaryotic expression | ILKPEMIEKLNEQMNLELYSSLLYQQMSAWCSYHTFEGAAAFLRRHAQEEMTHMQRLFDYLTDTGNLPRINTVESPFAEYSSLDELFQETYKHEQLITQKINELAHAAMTNQDYPTFNFLQWYVSEQHEEEKLFKSIIDKLSLAGKSGEGLYFIDKELSTLDTQN | This study |
| Eukaryotic-optimized H7HA1 with the removal of the signal peptide portion | DKICLGHHAVSNGTKVNTLTERGVEVVNATETVERTNIPRICSKGKRTVDLGQCGLLGTITGPPQCDQFLEFSADLIIERREGSDVCYPGKFVNEEALRQILRESGGIDKEPMGFTYNGIRTNGVTSACRRSGSSFYAEMKWLLSNTDNAAFPQMTKSYKNTRESPAIIVWGIHHSVSTAEQTKLYGSGNKLVTVGSSNYQQSFVPSPGARPQVNGQSGRIDFHWLILNPNDTVTFSFNGAFIAPDRASFLRGKSMGIQSGVQVDANCEGDGGGSGGGSGGGSGGGSAHIVMVDAYKPTKHHHHHH | This study |
