# Supplementary material for: A glycoengineered yeast-derived HA1-ferritin nanoparticle vaccine confers protective immunity against H7 avian influenza in chickens
Source: Vet Res. 2026 Oct 29;57:190. doi: 10.1186/s13567-026-01867-y (PMC13721490; doi:10.1186/s13567-026-01867-y)
Supplement: Supplementary file 8 [file 13567_2026_1867_MOESM17_ESM.docx]

**Title**: A glycoengineered yeast-derived HA1-ferritin nanoparticle vaccine confers protective immunity against H7 avian influenza in chickens

**Authors**: Yutong Liu, Yunlin Shao, Bo Su, Peiqi Liu, Yifeng Huang, Han Zheng, Siqi Xu, Yishan Zhao, Qianyu Li, Ming Liao, Saixiang Feng

**Additional file 17.** Primers used in this study.

| Primer name | Primer sequences | Characteristic(s) |
| --- | --- | --- |
| 5-α factor-F | TACTATTGCCAGCATTGCTGC | 1297 bp |
| 3-AOX1-R | GGCAAATGGCATTCTGACAT |  |
| T7-F | TAATACGACTCACTATAGGG | 1128 bp |
| T7-R | TGCTAGTTATTGCTCAGCGG |  |
