# Supplementary material for: A glycoengineered yeast-derived HA1-ferritin nanoparticle vaccine confers protective immunity against H7 avian influenza in chickens
Source: Vet Res. 2026 Oct 29;57:190. doi: 10.1186/s13567-026-01867-y (PMC13721490; doi:10.1186/s13567-026-01867-y)
Supplement: Supplementary file 9 [file 13567_2026_1867_MOESM18_ESM.docx]

**Title**: A glycoengineered yeast-derived HA1-ferritin nanoparticle vaccine confers protective immunity against H7 avian influenza in chickens

**Authors**: Yutong Liu, Yunlin Shao, Bo Su, Peiqi Liu, Yifeng Huang, Han Zheng, Siqi Xu, Yishan Zhao, Qianyu Li, Ming Liao, Saixiang Feng

**Additional file 18.** Strains and plasmids used in this study.

| Name | Characteristic(s) | Source |
| --- | --- | --- |
| *E. coli* DH5α | F^-^, φ80d/*lacZ*ΔM15, Δ (*lacZYA-argF*) U169  *recA*1 *endA*1 *hsdR*17 | Laboratory collection |
| *E. coli* BL21 (DE3) | F^-^, *ompT hsdS_B_* (r_B_^-^, m_B_^-^) *gal dcm* (DE3) | Laboratory collection |
| *P. pastoris* X-33 | Wild-type strain | Laboratory collection |
| *P. pastoris* SuperMan5-10 | Δ*OCH1*, GAP-mannosidaseHDEL | BioGrammatics |
| Mut^S^ pPICZαA-H7HA1-Spytag | The *P. pastoris* Superman5 strain containing the recombinant plasmid pPICZαA-H7HA1-Spytag, with low methanol utilization efficiency. | This study |
| Mut^+^ pPICZαA-H7HA1-Spytag | The *P. pastoris* Superman5 strain containing the recombinant plasmid pPICZαA-H7HA1-Spytag, with high methanol utilization efficiency. | This study |
| X33 pPICZαA-H7HA1-Spytag | The *P. pastoris* X-33 strain containing the recombinant plasmid pPICZαA-H7HA1-Spytag, with high methanol utilization efficiency. | This study |
