# Supplementary material for: A glycoengineered yeast-derived HA1-ferritin nanoparticle vaccine confers protective immunity against H7 avian influenza in chickens
Source: Vet Res. 2026 Oct 29;57:190. doi: 10.1186/s13567-026-01867-y (PMC13721490; doi:10.1186/s13567-026-01867-y)
Supplement: Supplementary file 10 [file 13567_2026_1867_MOESM1_ESM.docx]

**Title**: A glycoengineered yeast-derived HA1-ferritin nanoparticle vaccine confers protective immunity against H7 avian influenza in chickens

**Authors**: Yutong Liu, Yunlin Shao, Bo Su, Peiqi Liu, Yifeng Huang, Han Zheng, Siqi Xu, Yishan Zhao, Qianyu Li, Ming Liao, Saixiang Feng

**Additional file 1.** SDS-PAGE identification of the recombinant protein HA1-X33 expressed by the *K. phaffii* expression system. “M” denotes the 180 kDa molecular weight marker, and lane 1 corresponds to the HA1-X33. HA1-X33 exhibited diffuse bands between 70 and 100 kDa.
