# Supplementary material for: A glycoengineered yeast-derived HA1-ferritin nanoparticle vaccine confers protective immunity against H7 avian influenza in chickens
Source: Vet Res. 2026 Oct 29;57:190. doi: 10.1186/s13567-026-01867-y (PMC13721490; doi:10.1186/s13567-026-01867-y)
Supplement: Supplementary file 11 [file 13567_2026_1867_MOESM2_ESM.docx]

**Title**: A glycoengineered yeast-derived HA1-ferritin nanoparticle vaccine confers protective immunity against H7 avian influenza in chickens

**Authors**: Yutong Liu, Yunlin Shao, Bo Su, Peiqi Liu, Yifeng Huang, Han Zheng, Siqi Xu, Yishan Zhao, Qianyu Li, Ming Liao, Saixiang Feng

**Additional file 2.** Analysis of *N*-linked glycoforms and glycosylation sites of HA1-Mut^+^

| Type | Position | Enzyme | Peptide | Glycans NHFAGNa | Modification Type | Observed (M+H) | Calc. mass (M+H) | Mass error (ppm) | Starting position | Score | Intensity |
| --- | --- | --- | --- | --- | --- | --- | --- | --- | --- | --- | --- |
| *N*-Glyco | 12 | TA | K.IC[+57.021]LGHHAVSN[+1216.423]GTK.V | HexNAc(2)Hex(5) | N[+1216] | 2610.1250 | 2610.1234 | 0.6 | 3 | 1044.25 | 7941300000 |
|  |  | C | L.GHHAVSN[+1540.529]GTKVNTL.T | HexNAc(2)Hex(7) | N[+1541] | 2975.2728 | 2975.2733 | -0.2 | 6 | 910.72 | 1253500000 |
|  |  | TA | -.DKIC[+57.021]LGHHAVSN[+1216.423]GTK.V | HexNAc(2)Hex(5) | N[+1216] | 2853.2539 | 2853.2453 | 3.0 | 1 | 851.93 | 282890000 |
|  |  | TG | D.KIC[+57.021]LGHHAVSN[+1216.423]GTK.V | HexNAc(2)Hex(5) | N[+1216] | 2738.2207 | 2738.2183 | 0.9 | 2 | 420.68 | 95721000 |
|  |  | TG | K.IC[+57.021]LGHHAVSN[+2188.740]GTKVNTLTER.G | HexNAc(2)Hex(11) | N[+2189] | 4395.8697 | 4395.8748 | -1.2 | 3 | 339.74 | 56679000 |
|  | 28 | TA | R.GVEVVN[+1216.423]ATETVER.T | HexNAc(2)Hex(5) | N[+1216] | 2619.1393 | 2619.1401 | -0.3 | 23 | 862.16 | 32465000000 |
|  |  | TG | E.VVN[+1054.370]ATETVER.T | HexNAc(2)Hex(4) | N[+1054] | 2171.9510 | 2171.9548 | -1.8 | 26 | 621.54 | 34396000 |
|  |  | TG | E.VVN[+1864.634]ATETVE.R | HexNAc(2)Hex(9) | N[+1865] | 2826.1181 | 2826.1178 | 0.1 | 26 | 593.53 | 158420000 |
|  |  | TG | E.VVN[+730.264]ATE.T | HexNAc(2)Hex(2) | N[+730] | 1362.5899 | 1362.5894 | 0.4 | 26 | 514.20 | 565990000 |
|  |  | TG | R.GVEVVN[+1216.423]ATE.T | HexNAc(2)Hex(5) | N[+1216] | 2133.8759 | 2133.8803 | -2.1 | 23 | 448.63 | 303650000 |
|  |  | TA | R.GVEVVN[+1216.423]ATETVERTNIPR.I | HexNAc(2)Hex(5) | N[+1216] | 3200.4566 | 3200.4687 | -3.8 | 23 | 391.99 | 106360000 |
|  | 231 | C | L.ILNPN[+1702.581]DTVTF.S | HexNAc(2)Hex(8) | N[+1703] | 2836.1676 | 2836.1650 | 0.9 | 227 | 603.72 | 2216600000 |
|  |  | C | W.LILNPN[+1054.370]DTVTF.S | HexNAc(2)Hex(4) | N[+1054] | 2301.0372 | 2301.0378 | -0.3 | 226 | 601.89 | 790000000 |
|  |  | TA | R.IDFHWLILNPN[+1216.423]DTVTFSFNGAFIAPDR.A | HexNAc(2)Hex(5) | N[+1216] | 4337.9749 | 4336.9806 | -2.1 | 221 | 568.91 | 68992000 |
|  |  | TA | R.IDFHWLILNPN[+1054.370].D | HexNAc(2)Hex(4) | N[+1054] | 2436.1004 | 2436.0963 | 1.7 | 221 | 471.67 | 19623000 |
|  |  | C | L.NPN[+1216.423]DTVTF.S | HexNAc(2)Hex(5) | N[+1216] | 2123.8393 | 2123.8385 | 0.4 | 229 | 353.62 | 291060000 |
|  |  | C | F.HWLILNPN[+1216.423]DTVTF.S | HexNAc(2)Hex(5) | N[+1216] | 2786.2260 | 2786.2289 | -1.0 | 224 | 347.67 | 35752000 |
|  |  | TA | I.DFHWLILNPN[+203.079].D | HexNAc(1) | N[+203] | 1471.7218 | 1471.7216 | 0.2 | 222 | 207.12 | 297780000 |

*^a^*Type indicates the type of glycosylation modification. *^b^*Position refers to the location of the glycosylation site within the protein sequence. *^c^*Peptide denotes the peptide sequence. *^d^*Glycans NHFAGNa represents the glycan structure attached to the peptide. *^e^*Modification Type(s) specifies the molecular weight of the glycan. *^f^*Observed (M+H) corresponds to the experimentally determined molecular mass of the peptide. *^g^*Calc. mass (M+H) indicates the theoretical molecular mass of the peptide. *^h^*Mass error (ppm) shows the deviation between observed and calculated masses. *^i^*Score represents the confidence score of peptide identification. *^j^*Intensity reflects the relative abundance of the peptide.
