# Supplementary material for: A glycoengineered yeast-derived HA1-ferritin nanoparticle vaccine confers protective immunity against H7 avian influenza in chickens
Source: Vet Res. 2026 Oct 29;57:190. doi: 10.1186/s13567-026-01867-y (PMC13721490; doi:10.1186/s13567-026-01867-y)
Supplement: Supplementary file 12 [file 13567_2026_1867_MOESM3_ESM.docx]

**Title**: A glycoengineered yeast-derived HA1-ferritin nanoparticle vaccine confers protective immunity against H7 avian influenza in chickens

**Authors**: Yutong Liu, Yunlin Shao, Bo Su, Peiqi Liu, Yifeng Huang, Han Zheng, Siqi Xu, Yishan Zhao, Qianyu Li, Ming Liao, Saixiang Feng

**Additional file 3**. Analysis of *O*-linked glycoforms and glycosylation sites of HA1-Mut^+^

| Type | Position | Enzyme | Peptide | Glycans NHFAGNa | Modification Type | Observed (M+H) | Calc. mass (M+H) | Mass error (ppm) | Starting position | Score | Intensity |
| --- | --- | --- | --- | --- | --- | --- | --- | --- | --- | --- | --- |
| *O*-Glyco | 11 | TA | K.IC[+57.021]LGHHAVS[+1620.528]NGTK.V | Hex(10) | S[+1621] | 3014.2034 | 3014.2287 | -8.4 | 3 | 359.50 | 160580000 |
|  |  | TG | -.DKIC[+57.021]LGHHAVS[+972.317]NGTK.V | Hex(6) | S[+972] | 2609.1001 | 2609.1393 | -15.0 | 1 | 224.52 | 2402800000 |
|  |  | C | L.GHHAVS[+1620.528]NGTKVNTL.T | Hex(10) | S[+1621] | 3055.2397 | 3055.2730 | -10.9 | 6 | 124.68 | 76136000 |
|  | 14 | TA | K.IC[+57.021]LGHHAVSNGT[+1296.423]K.V | Hex(8) | T[+1296] | 2690.0906 | 2690.1231 | -12.1 | 3 | 530.84 | 964670000 |
|  |  | C | L.GHHAVSNGT[+1296.423]KVNTL.T | Hex(8) | T[+1296] | 2731.1357 | 2731.1674 | -11.6 | 6 | 484.13 | 120230000 |
|  | 18 | TA | K.VNT[+324.106]LTER.G | Hex(2) | T[+324] | 1156.5700 | 1156.5580 | 10.4 | 16 | 241.49 | 19380000 |
|  | 20 | TA | K.VNTLT[+648.211]ER.G | Hex(4) | T[+648] | 1480.6631 | 1480.6636 | -0.3 | 16 | 523.11 | 3684500000 |
|  |  | TG | K.VNTLT[+648.211]E.R | Hex(4) | T[+648] | 1324.5647 | 1324.5625 | 1.7 | 16 | 445.56 | 41749000 |
|  | 30 | TA | R.GVEVVNAT[+324.106]ETVER.T | Hex(2) | T[+324] | 1726.8235 | 1726.8229 | 0.4 | 23 | 606.41 | 102560000 |
|  |  | TA | K.VNTLTERGVEVVNAT[+162.053]ETVER.T | Hex(1) | T[+162] | 2378.2502 | 2378.2045 | 19.2 | 16 | 185.89 | 46457000 |
|  |  | TG | E.VVNAT[+1458.475]E.T | Hex(9) | T[+1458] | 2090.7699 | 2090.8004 | -14.6 | 26 | 172.01 | 10147000 |
|  | 32 | TG | E.VVNATET[+404.072]VER.T | Hex(2)Phospho(1) | T[+404] | 1521.6340 | 1521.6568 | -15.0 | 26 | 269.80 | 18504000 |
|  | 36 | TG | E.RT[+324.106]NIPR.I | Hex(2) | T[+324] | 1080.5525 | 1080.5531 | -0.6 | 35 | 466.87 | 447250000 |
|  |  | TA | R.T[+324.106]NIPRIC[+57.021]SK.G | Hex(2) | T[+324] | 1412.6937 | 1412.6937 | -0.1 | 36 | 361.36 | 11933000 |
|  |  | TA | R.T[+162.053]NIPR.I | Hex(1) | T[+162] | 762.3991 | 762.3992 | -0.1 | 36 | 360.00 | 50990000 |
|  | 48 | TG | K.GKRT[+810.264]VDLGQC[+57.021]GLLGTITGPPQC[+57.021]DQFLE.F | Hex(5) | T[+810] | 3770.7566 | 3770.7246 | 8.5 | 45 | 146.29 | 312150000 |
|  |  | TG | R.T[+1620.528]VDLGQC[+57.021]GLLGTITGPPQC[+57.021]DQFLE.F | Hex(10) | T[+1621] | 4239.7751 | 4239.7712 | 0.9 | 48 | 111.12 | 14128000 |
|  | 59 | TG | R.TVDLGQC[+57.021]GLLGT[+324.106]ITGPPQC[+57.021]DQFLE.F | Hex(2) | T[+324] | 2943.3530 | 2943.3486 | 1.5 | 48 | 180.93 | 26954000 |
|  | 61 | C | L.LGTIT[+162.053]GPPQC[+57.021]DQF.L | Hex(1) | T[+162] | 1595.7524 | 1595.7258 | 16.7 | 57 | 341.64 | 10013000 |
|  |  | C | L.GTIT[+324.106]GPPQC[+57.021]DQFL.E | Hex(2) | T[+324] | 1757.8124 | 1757.7786 | 19.2 | 58 | 254.50 | 35077000 |
|  |  | C | L.GTIT[+162.053]GPPQC[+57.021]DQF.L | Hex(1) | T[+162] | 1482.6701 | 1482.6417 | 19.2 | 58 | 186.32 | 44184000 |
|  | 73 | TG | E.FS[+324.106]ADLIIER.R | Hex(2) | S[+324] | 1387.6844 | 1387.6839 | 0.4 | 72 | 544.40 | 212780000 |
|  |  | C | F.S[+162.053]ADLIIERREGSDVC[+57.021]YPGKF.V | Hex(1) | S[+162] | 2474.2056 | 2474.1868 | 7.6 | 73 | 155.03 | 117360000 |
|  | 84 | TG | R.EGS[+162.053]DVC[+57.021]YPGK.F | Hex(1) | S[+162] | 1273.5247 | 1273.5253 | -0.4 | 82 | 489.82 | 32972000 |
|  |  | C | F.SADLIIERREGS[+486.158]DVC[+57.021]YPGKFVNEEAL.R | Hex(3) | S[+486] | 3453.6097 | 3453.6101 | -0.1 | 73 | 280.39 | 35402000 |
|  |  | TG | R.REGS[+486.158]DVC[+57.021]YPGK.F | Hex(3) | S[+486] | 1753.7576 | 1753.7320 | 14.6 | 81 | 172.45 | 19927000 |
|  |  | C | F.SADLIIERREGS[+324.106]DVC[+57.021]YPGKFVNEEALRQIL.R | Hex(2) | S[+324] | 3801.8510 | 3801.8851 | -9.0 | 73 | 126.49 | 754360000 |
|  |  | C | F.SADLIIERREGS[+162.053]DVC[+57.021]YPGKF.V | Hex(1) | S[+162] | 2474.2107 | 2474.1868 | 9.7 | 73 | 110.28 | 18259000 |
|  | 105 | C | L.RES[+162.053]GGIDKEPM[+15.995]GF.T | Hex(1) | S[+162] | 1600.7154 | 1600.7159 | -0.3 | 103 | 330.49 | 57043000 |
|  |  | TG | E.S[+162.053]GGIDKEPMGFTYNGIR.T | Hex(1) | S[+162] | 2003.9017 | 2003.9379 | -18.0 | 105 | 182.31 | 173190000 |
|  |  | TG | R.ES[+972.317]GGIDK.E | Hex(6) | S[+972] | 1677.6911 | 1677.6583 | 19.5 | 104 | 165.54 | 54925000 |
|  |  | C | L.RQILRES[+162.053]GGIDKEPMGF.T | Hex(1) | S[+162] | 2095.0449 | 2095.0488 | -1.9 | 99 | 122.61 | 22146000 |
|  |  | TG | R.QILRES[+972.317]GGIDKEPM[+15.995]GFTYNGIR.T | Hex(6) | S[+972] | 3469.5305 | 3469.5673 | -10.6 | 100 | 113.60 | 51885000 |
|  | 116 | TA | R.ESGGIDKEPMGFT[+162.053]YNGIR.T | Hex(1) | T[+162] | 2132.9814 | 2132.9805 | 0.4 | 104 | 709.59 | 35203000 |
|  |  | TG | E.SGGIDKEPMGFT[+162.053]YNGIR.T | Hex(1) | T[+162] | 2003.9012 | 2003.9379 | -18.3 | 105 | 241.61 | 519250000 |
|  | 134 | TA | R.SGS[+324.106]SFYAEM[+15.995]K.W | Hex(2) | S[+324] | 1446.5823 | 1446.5828 | -0.4 | 132 | 430.34 | 112440000 |
|  | 135 | TA | R.SGSS[+324.106]FYAEMK.W | Hex(2) | S[+324] | 1430.5893 | 1430.5879 | 0.9 | 132 | 516.26 | 521550000 |
|  | 145 | TG | K.WLLS[+162.053]NTDNAAFPQMTK.S | Hex(1) | S[+162] | 1998.9205 | 1998.9477 | -13.6 | 142 | 408.70 | 121760000 |
|  |  | TG | E.MKWLLS[+972.317]NTDNAAFPQMTK.S | Hex(6) | S[+972] | 3068.4056 | 3068.3473 | 19.0 | 140 | 213.39 | 65118000 |
|  | 147 | TG | K.WLLSNT[+162.053]DNAAFPQM[+15.995]TK.S | Hex(1) | T[+162] | 2014.9813 | 2014.9426 | 19.2 | 142 | 400.80 | 235850000 |
|  | 156 | TG | K.WLLSNTDNAAFPQMT[+162.053]K.S | Hex(1) | T[+162] | 1998.9481 | 1998.9477 | 0.2 | 142 | 465.23 | 235740000 |
|  |  | C | W.LLSNTDNAAFPQMT[+162.053]KSY.K | Hex(1) | T[+162] | 2062.9689 | 2062.9638 | 2.5 | 143 | 313.15 | 53693000 |
|  |  | TA | T.DNAAFPQMT[+162.053]K.S | Hex(1) | T[+162] | 1284.5788 | 1284.5776 | 0.9 | 148 | 221.10 | 78648000 |
|  | 162 | C | Y.KNT[+162.053]RESPAIIVW.G | Hex(1) | T[+162] | 1575.8385 | 1575.8377 | 0.5 | 160 | 398.01 | 1057500000 |
|  | 165 | TG | R.ES[+162.053]PAIIVWGIHHSVSTAEQTK.L | Hex(1) | S[+162] | 2452.2327 | 2452.2354 | -1.1 | 164 | 995.13 | 4995700000 |
|  |  | TG | R.ES[+162.053]PAIIVWGIHHSVSTAE.Q | Hex(1) | S[+162] | 2095.0324 | 2095.0342 | -0.9 | 164 | 671.87 | 3525400000 |
|  |  | TG | E.S[+162.053]PAIIVWGIHHSVSTAEQTK.L | Hex(1) | S[+162] | 2323.1707 | 2323.1929 | -9.6 | 165 | 409.16 | 303780000 |
|  |  | TG | E.S[+404.072]PAIIVWGIHHSVSTAE.Q | Hex(2)Phospho(1) | S[+404] | 2208.0346 | 2208.0108 | 10.8 | 165 | 188.71 | 91719000 |
|  |  | C | Y.KNTRES[+162.053]PAIIVW.G | Hex(1) | S[+162] | 1575.8384 | 1575.8377 | 0.5 | 160 | 160.87 | 10113000 |
|  | 176 | TA | R.ESPAIIVWGIHHS[+324.106]VSTAEQTK.L | Hex(2) | S[+324] | 2614.3393 | 2614.2883 | 19.5 | 164 | 958.96 | 480690000 |
|  |  | TG | E.SPAIIVWGIHHS[+162.053]VSTAEQTK.L | Hex(1) | S[+162] | 2323.1869 | 2323.1929 | -2.5 | 165 | 920.90 | 131010000 |
|  |  | TA | R.ESPAIIVWGIHHS[+162.053]VSTAEQTKLYGSGNK.L | Hex(1) | S[+162] | 3171.6565 | 3171.5957 | 19.2 | 164 | 743.36 | 30830000 |
|  |  | TG | R.ESPAIIVWGIHHS[+162.053]VSTAE.Q | Hex(1) | S[+162] | 2095.0366 | 2095.0342 | 1.1 | 164 | 563.76 | 594650000 |
|  |  | TA | K.NTRESPAIIVWGIHHS[+162.053]VSTAEQTK.L | Hex(1) | S[+162] | 2823.4765 | 2823.4272 | 17.5 | 161 | 518.94 | 41458000 |
|  |  | C | W.GIHHS[+324.106]VSTAEQTKL.Y | Hex(2) | S[+324] | 1831.9158 | 1831.8920 | 13.0 | 172 | 154.22 | 19566000 |
|  |  | TG | E.SPAIIVWGIHHS[+404.072]VSTAE.Q | Hex(2)Phospho(1) | S[+404] | 2208.0277 | 2208.0108 | 7.7 | 165 | 109.44 | 231610000 |
|  | 178 | C | W.GIHHSVS[+162.053]TAEQTKL.Y | Hex(1) | S[+162] | 1669.8384 | 1669.8392 | -0.5 | 172 | 816.35 | 8122600000 |
|  |  | C | W.GIHHSVS[+162.053]TAEQTKLY.G | Hex(1) | S[+162] | 1832.9031 | 1832.9025 | 0.3 | 172 | 806.56 | 1003300000 |
|  |  | TG | R.ESPAIIVWGIHHSVS[+162.053]TAE.Q | Hex(1) | S[+162] | 2095.0388 | 2095.0342 | 2.2 | 164 | 555.82 | 3525400000 |
|  |  | TA | R.ESPAIIVWGIHHSVS[+162.053]TAEQTK.L | Hex(1) | S[+162] | 2452.2653 | 2452.2354 | 12.2 | 164 | 398.08 | 332670000 |
|  | 179 | TG | R.ESPAIIVWGIHHSVST[+162.053]AEQTK.L | Hex(1) | T[+162] | 2452.2695 | 2452.2354 | 13.9 | 164 | 785.90 | 1518600000 |
|  |  | TG | R.ESPAIIVWGIHHSVST[+162.053]AE.Q | Hex(1) | T[+162] | 2095.0403 | 2095.0342 | 2.9 | 164 | 260.82 | 3525400000 |
|  |  | C | W.GIHHSVST[+324.106]AEQTKL.Y | Hex(2) | T[+324] | 1831.9197 | 1831.8920 | 15.1 | 172 | 97.77 | 16506000 |
|  | 183 | TA | R.ESPAIIVWGIHHSVSTAEQT[+162.053]K.L | Hex(1) | T[+162] | 2452.2366 | 2452.2354 | 0.5 | 164 | 1013.24 | 1106100000 |
|  |  | C | W.GIHHSVSTAEQT[+162.053]KL.Y | Hex(1) | T[+162] | 1669.8072 | 1669.8392 | -19.1 | 172 | 185.96 | 28940000 |
|  | 188 | TG | K.LYGS[+324.106]GNKLVTVGSSNYQQSFVPSPGAR.P | Hex(2) | S[+324] | 3137.5862 | 3137.5273 | 18.8 | 185 | 339.50 | 292720000 |
|  | 194 | C | Y.GSGNKLVT[+162.053]VGSSNY.Q | Hex(1) | T[+162] | 1544.7444 | 1544.7439 | 0.4 | 187 | 707.80 | 1088700000 |
|  | 197 | TA | K.LVTVGS[+162.053]SNYQQSFVPSPGAR.P | Hex(1) | S[+162] | 2256.1198 | 2256.1143 | 2.4 | 192 | 683.57 | 270690000 |
|  | 198 | TG | K.LVTVGSS[+324.106]NYQQSFVPSPGAR.P | Hex(2) | S[+324] | 2418.1637 | 2418.1671 | -1.4 | 192 | 565.23 | 134630000 |
|  |  | TA | K.LYGSGNKLVTVGSS[+324.106]NYQQSFVPSPGAR.P | Hex(2) | S[+324] | 3137.5716 | 3137.5273 | 14.1 | 185 | 190.16 | 46667000 |
|  | 203 | TA | K.LVTVGSSNYQQS[+162.053]FVPSPGAR.P | Hex(1) | S[+162] | 2256.1194 | 2256.1143 | 2.3 | 192 | 468.62 | 60492000 |
|  | 207 | TG | K.LVTVGSSNYQQSFVPS[+162.053]PGAR.P | Hex(1) | S[+162] | 2256.1095 | 2256.1143 | -2.1 | 192 | 845.70 | 1500700000 |
|  | 233 | C | W.LILNPNDT[+1458.475]VTF.S | Hex(9) | T[+1458] | 2705.1089 | 2705.1432 | -12.7 | 226 | 208.49 | 45707000 |
|  |  | C | L.ILNPNDT[+1458.475]VTF.S | Hex(9) | T[+1458] | 2592.0286 | 2592.0591 | -11.8 | 227 | 119.89 | 68139000 |
|  | 235 | C | W.LILNPNDTVT[+1458.475]F.S | Hex(9) | T[+1458] | 2705.1151 | 2705.1432 | -10.4 | 226 | 379.97 | 110210000 |
|  |  | C | L.ILNPNDTVT[+1458.475]F.S | Hex(9) | T[+1458] | 2592.0304 | 2592.0591 | -11.1 | 227 | 371.45 | 185080000 |
|  | 237 | C | F.S[+648.211]FNGAF.I | Hex(4) | S[+648] | 1290.5157 | 1290.4995 | 12.6 | 237 | 182.14 | 92734000 |
|  | 249 | C | F.SFNGAFIAPDRAS[+162.053]F.L | Hex(1) | S[+162] | 1661.7824 | 1661.7806 | 1.1 | 237 | 264.18 | 19589000 |
|  |  | C | F.IAPDRAS[+162.053]FL.R | Hex(1) | S[+162] | 1151.6048 | 1151.5943 | 9.1 | 243 | 241.22 | 264130000 |
|  | 255 | TG | K.S[+162.053]M[+15.995]GIQSGVQVDANC[+57.021]E.G | Hex(1) | S[+162] | 1772.7333 | 1772.7313 | 1.1 | 255 | 283.27 | 68127000 |
|  |  | TA | K.S[+162.053]MGIQSGVQV.D | Hex(1) | S[+162] | 1167.5578 | 1167.5562 | 1.4 | 255 | 209.43 | 209050000 |
|  | 299 | TA | V.DAYKPT[+162.053]K.H | Hex(1) | T[+162] | 984.4892 | 984.4884 | 0.8 | 294 | 383.50 | 43963000 |

*^a^*Type indicates the type of glycosylation modification. *^b^*Position refers to the location of the glycosylation site within the protein sequence. *^c^*Peptide denotes the peptide sequence. *^d^*Glycans NHFAGNa represents the glycan structure attached to the peptide. *^e^*Modification Type(s) specifies the molecular weight of the glycan. *^f^*Observed (M+H) corresponds to the experimentally determined molecular mass of the peptide. *^g^*Calc. mass (M+H) indicates the theoretical molecular mass of the peptide. *^h^*Mass error (ppm) shows the deviation between observed and calculated masses. *^i^*Score represents the confidence score of peptide identification. *^j^*Intensity reflects the relative abundance of the peptide.
