# Supplementary material for: A glycoengineered yeast-derived HA1-ferritin nanoparticle vaccine confers protective immunity against H7 avian influenza in chickens
Source: Vet Res. 2026 Oct 29;57:190. doi: 10.1186/s13567-026-01867-y (PMC13721490; doi:10.1186/s13567-026-01867-y)
Supplement: Supplementary file 13 [file 13567_2026_1867_MOESM4_ESM.docx]

**Title**: A glycoengineered yeast-derived HA1-ferritin nanoparticle vaccine confers protective immunity against H7 avian influenza in chickens

**Authors**: Yutong Liu, Yunlin Shao, Bo Su, Peiqi Liu, Yifeng Huang, Han Zheng, Siqi Xu, Yishan Zhao, Qianyu Li, Ming Liao, Saixiang Feng

**Additional file 4.** Analysis of *N*-linked glycoforms and glycosylation sites of HA1-Mut^S^

| Type | Position | Enzyme | Peptide | Glycans NHFAGNa | Modification Type | Observed (M+H) | Calc. mass (M+H) | Mass error (ppm) | Starting position | Score | Intensity |
| --- | --- | --- | --- | --- | --- | --- | --- | --- | --- | --- | --- |
| *N*-Glyco | 12 | TA | K.IC[+57.021]LGHHAVSN[+1216.423]GTK.V | HexNAc(2)Hex(5) | N[+1216] | 2610.1260 | 2610.1234 | 1.0 | 3 | 772.63 | 224840000 |
|  |  | TG | -.DKIC[+57.021]LGHHAVSN[+1216.423]GTK.V | HexNAc(2)Hex(5) | N[+1216] | 2853.2803 | 2853.2453 | 12.3 | 1 | 310.12 | 19190000 |
|  | 28 | TA | R.GVEVVN[+1216.423]ATETVER.T | HexNAc(2)Hex(5) | N[+1216] | 2619.1420 | 2619.1401 | 0.7 | 23 | 737.84 | 2308300000 |
|  |  | TG | E.VVN[+203.079]ATETVER.T | HexNAc(1) | N[+203] | 1320.6797 | 1320.6642 | 11.8 | 26 | 509.63 | 300810000 |
|  |  | TG | R.GVEVVN[+1216.423]ATETVE.R | HexNAc(2)Hex(5) | N[+1216] | 2463.0687 | 2463.0390 | 12.1 | 23 | 388.01 | 34998000 |
|  |  | TG | E.RGVEVVN[+1216.423]ATE.T | HexNAc(2)Hex(5) | N[+1216] | 2289.9977 | 2289.9814 | 7.1 | 22 | 195.89 | 69493000 |
|  | 231 | TG | R.IDFHWLILNPN[+1216.423]DTVTFSFNGAFIAPDR.A | HexNAc(2)Hex(5) | N[+1216] | 4337.0322 | 4336.9806 | 11.9 | 221 | 775.72 | 2340200000 |
|  |  | C | W.LILNPN[+1216.423]DTVTF.S | HexNAc(2)Hex(5) | N[+1216] | 2463.0924 | 2463.0906 | 0.7 | 226 | 651.41 | 718030000 |
|  |  | C | L.ILNPN[+1702.581]DTVTF.S | HexNAc(2)Hex(8) | N[+1703] | 2836.1637 | 2836.1650 | -0.5 | 227 | 591.30 | 2285300000 |
|  |  | TA | R.IDFHWLILNPN[+1216.423].D | HexNAc(2)Hex(5) | N[+1216] | 2598.1539 | 2598.1492 | 1.8 | 221 | 559.70 | 775630000 |
|  |  | TA | I.DFHWLILNPN[+1216.423].D | HexNAc(2)Hex(5) | N[+1216] | 2485.0631 | 2485.0651 | -0.8 | 222 | 418.64 | 64901000 |
|  |  | TG | K.LVTVGSSNYQQSFVPSPGARPQVNGQSGRIDFHWLILNPN[+1216.423]DTVTFSFNGAFIAPDRASFLR.G | HexNAc(2)Hex(5) | N[+1216] | 7908.8944 | 7909.8043 | 11.8 | 192 | 314.13 | 584760000 |
|  |  | C | F.HWLILNPN[+892.317]DTVTF.S | HexNAc(2)Hex(3) | N[+892] | 2462.0878 | 2462.1232 | -14.4 | 224 | 187.87 | 35215000 |
|  |  | C | L.NPN[+406.159]DTVTF.S | HexNAc(2) | N[+406] | 1313.5724 | 1313.5743 | -1.5 | 229 | 113.25 | 53859000 |
|  |  | TG | K.LVTVGSSNYQQSFVPSPGARPQVNGQSGRIDFHWLILNPN[+406.159]DTVTFSFNGAFIAPDR.A | HexNAc(2) | N[+406] | 6526.1682 | 6525.2175 | -8.1 | 192 | 95.96 | 173670000 |

*^a^*Type indicates the type of glycosylation modification. *^b^*Position refers to the location of the glycosylation site within the protein sequence. *^c^*Peptide denotes the peptide sequence. *^d^*Glycans NHFAGNa represents the glycan structure attached to the peptide. *^e^*Modification Type(s) specifies the molecular weight of the glycan. *^f^*Observed (M+H) corresponds to the experimentally determined molecular mass of the peptide. *^g^*Calc. mass (M+H) indicates the theoretical molecular mass of the peptide. *^h^*Mass error (ppm) shows the deviation between observed and calculated masses. *^i^*Score represents the confidence score of peptide identification. *^j^*Intensity reflects the relative abundance of the peptide.
