# Supplementary material for: A glycoengineered yeast-derived HA1-ferritin nanoparticle vaccine confers protective immunity against H7 avian influenza in chickens
Source: Vet Res. 2026 Oct 29;57:190. doi: 10.1186/s13567-026-01867-y (PMC13721490; doi:10.1186/s13567-026-01867-y)
Supplement: Supplementary file 14 [file 13567_2026_1867_MOESM5_ESM.docx]

**Title**: A glycoengineered yeast-derived HA1-ferritin nanoparticle vaccine confers protective immunity against H7 avian influenza in chickens

**Authors**: Yutong Liu, Yunlin Shao, Bo Su, Peiqi Liu, Yifeng Huang, Han Zheng, Siqi Xu, Yishan Zhao, Qianyu Li, Ming Liao, Saixiang Feng

**Additional file 5**. Analysis of *O*-linked glycoforms and glycosylation sites of HA1-Mut^S^

| Type | Position | Enzyme | Peptide | Glycans NHFAGNa | Modification Type | Observed (M+H) | Calc. mass (M+H) | Mass error (ppm) | Starting position | Score | Intensity |
| --- | --- | --- | --- | --- | --- | --- | --- | --- | --- | --- | --- |
| O-Glyco | 18 | TG | K.VNT[+648.211]LTER.G | Hex(4) | T[+648] | 1480.6833 | 1480.6636 | 13.3 | 16 | 196.81 | 15416000 |
|  |  | TA | K.VNT[+648.211]LTERGVEVVNATETVER.T | Hex(4) | T[+648] | 2864.3994 | 2864.3630 | 12.7 | 16 | 112.43 | 7778800 |
|  | 20 | TA | K.VNTLT[+324.106]ER.G | Hex(2) | T[+324] | 1156.5578 | 1156.5580 | -0.1 | 16 | 483.99 | 461560000 |
|  | 59 | TA | R.TVDLGQC[+57.021]GLLGT[+972.317]ITGPPQC[+57.021].D | Hex(6) | T[+972] | 2959.2562 | 2959.2793 | -7.8 | 48 | 118.26 | 39753000 |
|  | 61 | C | L.LGTIT[+162.053]GPPQC[+57.021]DQFLEF.S | Hex(1) | T[+162] | 1984.9213 | 1984.9208 | 0.2 | 57 | 430.79 | 62946000 |
|  |  | TA | V.DLGQC[+57.021]GLLGTIT[+324.106]GPPQC[+57.021].D | Hex(2) | T[+324] | 2110.9552 | 2110.9519 | 1.6 | 50 | 420.57 | 18205000 |
|  |  | C | L.GTIT[+162.053]GPPQC[+57.021]DQF.L | Hex(1) | T[+162] | 1482.6411 | 1482.6417 | -0.4 | 58 | 286.61 | 21706000 |
|  |  | C | L.LGTIT[+162.053]GPPQC[+57.021]DQF.L | Hex(1) | T[+162] | 1595.7566 | 1595.7258 | 19.3 | 57 | 241.53 | 18730000 |
|  |  | TG | R.TVDLGQC[+57.021]GLLGTIT[+162.053]GPPQC[+57.021]DQFLEFSADLIIER.R | Hex(1) | T[+162] | 3825.9234 | 3825.8561 | 17.6 | 48 | 177.91 | 225040000 |
|  |  | TA | V.DLGQC[+57.021]GLLGTIT[+648.211]GPPQC[+57.021]DQFLEFSADLIIER.R | Hex(4) | T[+648] | 4110.9014 | 4111.8985 | 1.5 | 50 | 18.38 | 110180000 |
|  | 73 | C | F.S[+162.053]ADLIIERREGSDVC[+57.021]YPGKF.V | Hex(1) | S[+162] | 2474.1891 | 2474.1868 | 0.9 | 73 | 341.45 | 124800000 |
|  | 84 | TG | R.REGS[+324.106]DVC[+57.021]YPGK.F | Hex(2) | S[+324] | 1591.6972 | 1591.6792 | 11.3 | 81 | 418.90 | 40355000 |
|  |  | TG | E.GS[+162.053]DVC[+57.021]YPGKFVNEEALR.Q | Hex(1) | S[+162] | 2102.9400 | 2102.9699 | -14.2 | 83 | 348.17 | 194520000 |
|  |  | C | F.SADLIIERREGS[+486.158]DVC[+57.021]YPGKFVNEEAL.R | Hex(3) | S[+486] | 3453.6053 | 3453.6101 | -1.4 | 73 | 217.67 | 90098000 |
|  |  | C | L.IIERREGS[+162.053]DVC[+57.021]YPGKF.V | Hex(1) | S[+162] | 2087.9774 | 2088.0066 | -14.0 | 77 | 89.91 | 26829000 |
|  | 105 | C | L.RES[+162.053]GGIDKEPMGF.T | Hex(1) | S[+162] | 1584.7234 | 1584.7210 | 1.5 | 103 | 444.26 | 311250000 |
|  |  | TG | R.QILRES[+972.317]GGIDKEPM[+15.995]GFTYNGIR.T | Hex(6) | S[+972] | 3469.5760 | 3469.5673 | 2.5 | 100 | 289.91 | 213980000 |
|  |  | TG | E.S[+162.053]GGIDKEPMGFTYNGIR.T | Hex(1) | S[+162] | 2003.9275 | 2003.9379 | -5.2 | 105 | 203.91 | 371780000 |
|  | 116 | TG | R.ESGGIDKEPMGFT[+162.053]YNGIR.T | Hex(1) | T[+162] | 2132.9990 | 2132.9805 | 8.7 | 104 | 653.66 | 348410000 |
|  |  | TG | E.PMGFT[+162.053]YNGIR.T | Hex(1) | T[+162] | 1317.5975 | 1317.6144 | -12.8 | 112 | 186.40 | 15084000 |
|  |  | TG | E.SGGIDKEPMGFT[+162.053]YNGIR.T | Hex(1) | T[+162] | 2003.9259 | 2003.9379 | -6.0 | 105 | 183.37 | 284500000 |
|  | 126 | TA | R.TNGVT[+162.053]SAC[+57.021]R.R | Hex(1) | T[+162] | 1127.5000 | 1127.4997 | 0.3 | 122 | 275.22 | 212960000 |
|  | 135 | TG | R.SGSS[+324.106]FYAEMK.W | Hex(2) | S[+324] | 1430.6070 | 1430.5879 | 13.3 | 132 | 450.09 | 1084000000 |
|  | 145 | TG | K.WLLS[+324.106]NTDNAAFPQM[+15.995]TK.S | Hex(2) | S[+324] | 2177.0216 | 2176.9955 | 12.0 | 142 | 334.01 | 39581000 |
|  |  | C | W.LLS[+162.053]NTDNAAFPQM[+15.995]TKSY.K | Hex(1) | S[+162] | 2078.9567 | 2078.9587 | -1.0 | 143 | 296.98 | 54479000 |
|  |  | TG | R.RSGSSFYAEMKWLLS[+162.053]NTDNAAFPQMTK.S | Hex(1) | S[+162] | 3242.5452 | 3242.5133 | 9.9 | 131 | 93.55 | 114030000 |
|  | 147 | C | W.LLSNT[+162.053]DNAAFPQMTKSY.K | Hex(1) | T[+162] | 2062.9641 | 2062.9638 | 0.2 | 143 | 485.16 | 221580000 |
|  | 156 | TG | K.WLLSNTDNAAFPQMT[+162.053]K.S | Hex(1) | T[+162] | 1998.9651 | 1998.9477 | 8.7 | 142 | 568.42 | 120240000 |
|  | 158 | C | F.PQMTKS[+324.106]Y.K | Hex(2) | S[+324] | 1178.5330 | 1178.5133 | 16.7 | 153 | 184.70 | 58905000 |
|  | 162 | TG | K.NT[+162.053]RESPAIIVWGIHHSVSTAEQTK.L | Hex(1) | T[+162] | 2823.4619 | 2823.4272 | 12.3 | 161 | 594.17 | 92755000 |
|  |  | C | Y.KNT[+162.053]RESPAIIVW.G | Hex(1) | T[+162] | 1575.8377 | 1575.8377 | 0.0 | 160 | 418.12 | 3223900000 |
|  |  | C | Y.KNT[+1944.634]RESPAIIVWGIHHSVSTAEQTKLY.G | Hex(12) | T[+1945] | 5011.1968 | 5010.2506 | -11.4 | 160 | 118.63 | 99303000 |
|  | 165 | TA | R.ES[+162.053]PAIIVWGIHHSVSTAEQTK.L | Hex(1) | S[+162] | 2452.2822 | 2452.2354 | 19.1 | 164 | 904.99 | 4036700000 |
|  |  | TG | K.NTRES[+162.053]PAIIVWGIHHSVSTAEQTK.L | Hex(1) | S[+162] | 2823.4612 | 2823.4272 | 12.0 | 161 | 595.12 | 92755000 |
|  |  | TG | R.ES[+162.053]PAIIVWGIHHSVSTAEQTKLYGSGNK.L | Hex(1) | S[+162] | 3171.6144 | 3171.5957 | 5.9 | 164 | 552.68 | 37574000 |
|  |  | TG | E.S[+404.072]PAIIVWGIHHSVSTAEQTK.L | Hex(2)Phospho(1) | S[+404] | 2565.2544 | 2565.2120 | 16.5 | 165 | 346.11 | 76301000 |
|  | 176 | TA | R.ESPAIIVWGIHHS[+162.053]VSTAEQTK.L | Hex(1) | S[+162] | 2452.2396 | 2452.2354 | 1.7 | 164 | 986.33 | 677650000 |
|  |  | TG | K.NTRESPAIIVWGIHHS[+162.053]VSTAEQTK.L | Hex(1) | S[+162] | 2823.4680 | 2823.4272 | 14.5 | 161 | 491.84 | 92755000 |
|  |  | TG | E.SPAIIVWGIHHS[+404.072]VSTAEQTK.L | Hex(2)Phospho(1) | S[+404] | 2565.2586 | 2565.2120 | 18.2 | 165 | 416.94 | 261160000 |
|  | 178 | C | W.GIHHSVS[+162.053]TAEQTKL.Y | Hex(1) | S[+162] | 1669.8398 | 1669.8392 | 0.4 | 172 | 750.65 | 3445200000 |
|  |  | C | W.GIHHSVS[+162.053]TAEQTKLY.G | Hex(1) | S[+162] | 1832.9219 | 1832.9025 | 10.6 | 172 | 286.33 | 163580000 |
|  |  | TG | R.ESPAIIVWGIHHSVS[+162.053]TAEQTK.L | Hex(1) | S[+162] | 2452.2833 | 2452.2354 | 19.5 | 164 | 198.20 | 113140000 |
|  | 183 | TA | R.ESPAIIVWGIHHSVSTAEQT[+162.053]K.L | Hex(1) | T[+162] | 2452.2368 | 2452.2354 | 0.5 | 164 | 962.45 | 576400000 |
|  |  | C | W.GIHHSVSTAEQT[+162.053]KL.Y | Hex(1) | T[+162] | 1669.8114 | 1669.8392 | -16.6 | 172 | 435.56 | 211730000 |
|  |  | C | W.GIHHSVSTAEQT[+162.053]KLY.G | Hex(1) | T[+162] | 1832.9203 | 1832.9025 | 9.7 | 172 | 217.70 | 210140000 |
|  |  | C | W.GIHHSVSTAEQT[+404.072]KLYGSGNKLVTVGSSNY.Q | Hex(2)Phospho(1) | T[+404] | 3438.6592 | 3438.5948 | 18.7 | 172 | 39.83 | 1071000000 |
|  | 194 | C | Y.GSGNKLVT[+162.053]VGSSNY.Q | Hex(1) | T[+162] | 1544.7425 | 1544.7439 | -0.9 | 187 | 611.32 | 3788800000 |
|  |  | TG | K.LVT[+162.053]VGSSNYQQSFVPSPGARPQVNGQSGRIDFHWLILNPNDTVTFSFNGAFIAPDR.A | Hex(1) | T[+162] | 6281.0252 | 6281.1115 | -13.7 | 192 | 94.19 | 189720000 |
|  |  | TG | E.QTKLYGSGNKLVT[+972.317]VGSSNYQQSFVPSPGAR.P | Hex(6) | T[+972] | 4144.0186 | 4142.9398 | 18.2 | 182 | 53.80 | 152640000 |
|  | 197 | TA | K.LVTVGS[+162.053]SNYQQSFVPSPGAR.P | Hex(1) | S[+162] | 2256.1152 | 2256.1143 | 0.4 | 192 | 632.41 | 88963000 |
|  | 198 | TA | K.LVTVGSS[+324.106]NYQQSFVPSPGAR.P | Hex(2) | S[+324] | 2418.1710 | 2418.1671 | 1.6 | 192 | 564.81 | 32341000 |
|  |  | TG | K.LYGSGNKLVTVGSS[+324.106]NYQQSFVPSPGAR.P | Hex(2) | S[+324] | 3137.5295 | 3137.5273 | 0.7 | 185 | 188.15 | 40342000 |
|  | 203 | TG | K.LVTVGSSNYQQS[+162.053]FVPSPGAR.P | Hex(1) | S[+162] | 2256.1416 | 2256.1143 | 12.1 | 192 | 461.20 | 62904000 |
|  | 207 | TG | K.LVTVGSSNYQQSFVPS[+162.053]PGAR.P | Hex(1) | S[+162] | 2256.1378 | 2256.1143 | 10.4 | 192 | 462.20 | 1278000000 |
|  |  | TG | K.LVTVGSSNYQQSFVPS[+162.053]PGARPQVNGQSGRIDFHWLILNPNDTVTFSFNGAFIAPDR.A | Hex(1) | S[+162] | 6282.0286 | 6281.1115 | -13.7 | 192 | 30.07 | 558230000 |
|  | 233 | C | W.LILNPNDT[+1944.634]VTF.S | Hex(12) | T[+1945] | 3191.2790 | 3191.3017 | -7.1 | 226 | 91.41 | 34313000 |
|  | 235 | C | L.ILNPNDTVT[+1620.528]F.S | Hex(10) | T[+1621] | 2754.0805 | 2754.1120 | -11.4 | 227 | 318.40 | 334070000 |
|  |  | C | W.LILNPNDTVT[+1620.528]F.S | Hex(10) | T[+1621] | 2867.1705 | 2867.1960 | -8.9 | 226 | 310.42 | 150900000 |
|  | 249 | TG | R.AS[+324.106]FLR.G | Hex(2) | S[+324] | 917.4578 | 917.4462 | 12.6 | 248 | 275.21 | 418330000 |
|  | 260 | TA | K.SMGIQS[+162.053]GVQV.D | Hex(1) | S[+162] | 1167.5583 | 1167.5562 | 1.8 | 255 | 379.33 | 64772000 |

*^a^*Type indicates the type of glycosylation modification. *^b^*Position refers to the location of the glycosylation site within the protein sequence. *^c^*Peptide denotes the peptide sequence. *^d^*Glycans NHFAGNa represents the glycan structure attached to the peptide. *^e^*Modification Type(s) specifies the molecular weight of the glycan. *^f^*Observed (M+H) corresponds to the experimentally determined molecular mass of the peptide. *^g^*Calc. mass (M+H) indicates the theoretical molecular mass of the peptide. *^h^*Mass error (ppm) shows the deviation between observed and calculated masses. *^i^*Score represents the confidence score of peptide identification. *^j^*Intensity reflects the relative abundance of the peptide.
