# Supplementary material for: A glycoengineered yeast-derived HA1-ferritin nanoparticle vaccine confers protective immunity against H7 avian influenza in chickens
Source: Vet Res. 2026 Oct 29;57:190. doi: 10.1186/s13567-026-01867-y (PMC13721490; doi:10.1186/s13567-026-01867-y)
Supplement: Supplementary file 15 [file 13567_2026_1867_MOESM6_ESM.docx]

**Title**: A glycoengineered yeast-derived HA1-ferritin nanoparticle vaccine confers protective immunity against H7 avian influenza in chickens

**Authors**: Yutong Liu, Yunlin Shao, Bo Su, Peiqi Liu, Yifeng Huang, Han Zheng, Siqi Xu, Yishan Zhao, Qianyu Li, Ming Liao, Saixiang Feng

**Additional file 6**. Site efficiency analysis of *O*-linked and *N*-linked glycosylation in HA1-Mut^+^

| Position | Type | Sum Intensity | Glycosylation efficiency (%) |
| --- | --- | --- | --- |
| 11 | O-Glyco | 43698486000 | 22.11 |
|  | non | 153949212200 |  |
| 14 | O-Glyco | 1280532000 | 0.65 |
|  | non | 196367166200 |  |
| 18 | O-Glyco | 12123098000 | 1.73 |
|  | non | 689623349100 |  |
| 20 | O-Glyco | 486135033100 | 70.44 |
|  | non | 203965932000 |  |
| 30 | O-Glyco | 923183000 | 0.16 |
|  | non | 588424979100 |  |
| 32 | O-Glyco | 18504000 | 0.00 |
|  | non | 480052545300 |  |
| 36 | O-Glyco | 599402700 | 18.03 |
|  | non | 2725470400 |  |
| 48 | O-Glyco | 326278000 | 0.74 |
|  | non | 43875765500 |  |
| 59 | O-Glyco | 56042000 | 0.08 |
|  | non | 72093448700 |  |
| 61 | O-Glyco | 99287000 | 0.14 |
|  | non | 72050203700 |  |
| 73 | O-Glyco | 615243000 | 0.18 |
|  | non | 344172828500 |  |
| 84 | O-Glyco | 5716860000 | 0.89 |
|  | non | 640049082600 |  |
| 105 | O-Glyco | 1336532000 | 0.20 |
|  | non | 656552259500 |  |
| 116 | O-Glyco | 8476695300 | 1.90 |
|  | non | 436898345000 |  |
| 134 | O-Glyco | 174630000 | 0.09 |
|  | non | 196530314000 |  |
| 135 | O-Glyco | 612769000 | 0.31 |
|  | non | 196092175000 |  |
| 145 | O-Glyco | 2092678300 | 0.62 |
|  | non | 333832582000 |  |
| 147 | O-Glyco | 343640000 | 0.11 |
|  | non | 311440915300 |  |
| 156 | O-Glyco | 418746000 | 0.06 |
|  | non | 685785897300 |  |
| 162 | O-Glyco | 5420917800 | 10.02 |
|  | non | 48676066200 |  |
| 165 | O-Glyco | 97198750700 | 7.10 |
|  | non | 1271160591800 |  |
| 176 | O-Glyco | 40252640800 | 2.38 |
|  | non | 1653140932100 |  |
| 178 | O-Glyco | 13643123000 | 0.81 |
|  | non | 1679750449900 |  |
| 179 | O-Glyco | 5590594000 | 0.33 |
|  | non | 1687802978900 |  |
| 183 | O-Glyco | 1904233000 | 0.15 |
|  | non | 1272825989700 |  |
| 188 | O-Glyco | 361821000 | 0.19 |
|  | non | 191314821400 |  |
| 194 | O-Glyco | 1088700000 | 0.10 |
|  | non | 1066367236500 |  |
| 197 | O-Glyco | 1941115000 | 0.18 |
|  | non | 1065514821500 |  |
| 198 | O-Glyco | 412614400 | 0.04 |
|  | non | 1067043322100 |  |
| 203 | O-Glyco | 78388000 | 0.01 |
|  | non | 839915359900 |  |
| 207 | O-Glyco | 3521880000 | 0.42 |
|  | non | 837242455900 |  |
| 233 | O-Glyco | 113846000 | 0.02 |
|  | non | 754846525500 |  |
| 235 | O-Glyco | 2319038600 | 0.31 |
|  | non | 752641332900 |  |
| 237 | O-Glyco | 92734000 | 0.18 |
|  | non | 51153566400 |  |
| 249 | O-Glyco | 283719000 | 0.04 |
|  | non | 668445084400 |  |
| 255 | O-Glyco | 345304000 | 0.69 |
|  | non | 49906960000 |  |
| 299 | O-Glyco | 62983200 | 6.60 |
|  | non | 891231000 |  |
| 12 | N-Glyco | 152055890200 | 76.93 |
|  | non | 45591808000 |  |
| 28 | N-Glyco | 584079683100 | 99.11 |
|  | non | 5268479000 |  |
| 231 | N-Glyco | 188907641600 | 24.97 |
|  | non | 567682532300 |  |
