# Supplementary material for: A glycoengineered yeast-derived HA1-ferritin nanoparticle vaccine confers protective immunity against H7 avian influenza in chickens
Source: Vet Res. 2026 Oct 29;57:190. doi: 10.1186/s13567-026-01867-y (PMC13721490; doi:10.1186/s13567-026-01867-y)
Supplement: Supplementary file 16 [file 13567_2026_1867_MOESM7_ESM.docx]

**Title**: A glycoengineered yeast-derived HA1-ferritin nanoparticle vaccine confers protective immunity against H7 avian influenza in chickens

**Authors**: Yutong Liu, Yunlin Shao, Bo Su, Peiqi Liu, Yifeng Huang, Han Zheng, Siqi Xu, Yishan Zhao, Qianyu Li, Ming Liao, Saixiang Feng

**Additional file 7**. Site efficiency analysis of *O*-linked and *N*-linked glycosylation in HA1-Mut^S^

| Position | Type | Sum Intensity | Glycosylation efficiency (%) |
| --- | --- | --- | --- |
| 18 | O-Glyco | 23194800 | 1.13 |
|  | non | 2036783000 |  |
| 20 | O-Glyco | 1783173000 | 86.56 |
|  | non | 276804800 |  |
| 59 | O-Glyco | 39753000 | 0.00 |
|  | non | 880173148000 |  |
| 61 | O-Glyco | 502529000 | 0.06 |
|  | non | 879710372000 |  |
| 73 | O-Glyco | 571030000 | 0.06 |
|  | non | 967433745900 |  |
| 84 | O-Glyco | 3276816000 | 0.24 |
|  | non | 1334559291100 |  |
| 105 | O-Glyco | 1645852000 | 0.11 |
|  | non | 1473387842700 |  |
| 116 | O-Glyco | 3289495700 | 0.44 |
|  | non | 736128352100 |  |
| 126 | O-Glyco | 212960000 | 7.71 |
|  | non | 2547724900 |  |
| 135 | O-Glyco | 1084000000 | 0.19 |
|  | non | 562239152100 |  |
| 145 | O-Glyco | 347442000 | 0.03 |
|  | non | 1063196805200 |  |
| 147 | O-Glyco | 221580000 | 0.02 |
|  | non | 1023992538000 |  |
| 156 | O-Glyco | 505180900 | 0.04 |
|  | non | 1310100921500 |  |
| 158 | O-Glyco | 58905000 | 0.02 |
|  | non | 251920030000 |  |
| 162 | O-Glyco | 25273825100 | 11.61 |
|  | non | 192496399200 |  |
| 165 | O-Glyco | 32425793000 | 1.71 |
|  | non | 1866832295600 |  |
| 176 | O-Glyco | 9767398000 | 0.38 |
|  | non | 2582552742300 |  |
| 178 | O-Glyco | 227416810000 | 8.77 |
|  | non | 2364903330300 |  |
| 183 | O-Glyco | 10891124000 | 0.42 |
|  | non | 2579157926300 |  |
| 194 | O-Glyco | 4131160000 | 0.30 |
|  | non | 1352893957800 |  |
| 197 | O-Glyco | 3049495000 | 0.22 |
|  | non | 1353975622800 |  |
| 198 | O-Glyco | 86704000 | 0.01 |
|  | non | 1356938413800 |  |
| 203 | O-Glyco | 81196000 | 0.01 |
|  | non | 924403455700 |  |
| 207 | O-Glyco | 3812350000 | 0.41 |
|  | non | 920284579700 |  |
| 233 | O-Glyco | 34313000 | 0.01 |
|  | non | 583577896960 |  |
| 235 | O-Glyco | 839818000 | 0.14 |
|  | non | 582772391960 |  |
| 249 | O-Glyco | 853453000 | 0.09 |
|  | non | 931623095300 |  |
| 260 | O-Glyco | 620682000 | 2.58 |
|  | non | 23480910300 |  |
| 12 | N-Glyco | 460111500 | 100.00 |
|  | non | 0 |  |
| 28 | N-Glyco | 22454924000 | 97.93 |
|  | non | 473865800 |  |
| 231 | N-Glyco | 237517412000 | 40.73 |
|  | non | 345600177360 |  |
