# Supplementary material for: A glycoengineered yeast-derived HA1-ferritin nanoparticle vaccine confers protective immunity against H7 avian influenza in chickens
Source: Vet Res. 2026 Oct 29;57:190. doi: 10.1186/s13567-026-01867-y (PMC13721490; doi:10.1186/s13567-026-01867-y)
Supplement: Supplementary file 17 [file 13567_2026_1867_MOESM8_ESM.docx]

**Title**: A glycoengineered yeast-derived HA1-ferritin nanoparticle vaccine confers protective immunity against H7 avian influenza in chickens

**Authors**: Yutong Liu, Yunlin Shao, Bo Su, Peiqi Liu, Yifeng Huang, Han Zheng, Siqi Xu, Yishan Zhao, Qianyu Li, Ming Liao, Saixiang Feng

**Additional file 8.** *O*-linked glycosylation analysis of HA1-Mut^S^ and HA1-Mut^+^. (A, B) Mass spectrometric analysis of *O*-linked glycosylation at T20 in HA1-Mut^S^ and HA1-Mut^+^. (C, D) Mass spectrometric analysis of *O*-linked glycosylation at T36 in both proteins. (E, F) Mass spectrometric analysis of *O*-linked glycosylation at T162 in HA1-Mut^S^ and HA1-Mut^+^. (G, H) Mass spectrometric analysis of *O*-linked glycosylation at S135 in both proteins. (I, J) Mass spectrometric analysis of *O*-linked glycosylation at S207 in HA1-Mut^S^ and HA1-Mut^+^. (K, L) Comparison of glycosylation efficiencies at T20, T36, T162, S135, and S207 for both proteins. HA1-Mut^S^ exhibits a simplified *O*-linked glycosylation profile compared to HA1-Mut^+^.
